# Supplementary material for: Topological phase transition in chiral graphene nanoribbons: from edge bands to end states
Source: Nat Commun. 2021 Sep 20;12:5538. doi: 10.1038/s41467-021-25688-z (PMC8452617; doi:10.1038/s41467-021-25688-z)
Supplement: Supplementary file 1 — Supplementary Information [file 41467_2021_25688_MOESM1_ESM.pdf]

Supplementary Information for: Topological phase transition in chiral graphene nanoribbons: from edge bands to end states

Jingcheng Li[1,2*], Sofia Sanz[3*], Nestor Merino-Díez[1,3*], Manuel Vilas-Varela[4*], Aran Garcia-Lekue[3,5], Martina Corso[2,3,5], Dimas G. de Oteyza [2,3,5△], Thomas Frederiksen[3,5△], Diego Peña[4△] & Jose Ignacio Pascual[1,5△]

[1]*CIC nanoGUNE-BRTA, 20018 Donostia-San Sebastián, Spain*
[2]*Centro de Física de Materiales MPC (CSIC-UPV/EHU), 20018 Donostia-San Sebastián, Spain*
[3] *Donostia International Physics Center (DIPC), 20018 Donostia-San Sebastián, Spain*
[4]*Centro Singular de Investigación en Química Biolóxica e Materiais Moleculares (CiQUS), and Departamento de Química Orgánica, Universidade de Santiago de Compostela, Spain*
[5]*Ikerbasque, Basque Foundation for Science, 48013 Bilbao, Spain* *August 3, 2021*

Contents

I Supplementary Methods 2

II Supplementary Notes 11

Supplementary Note 1 Structure of the family of chiral GNRs studied here 11

Supplementary Note 2 Complementary Tight Binding simulations 12

Supplementary Note 3 Determination of the topological phases of chiral GNRs 14

Supplementary Note 4 DFT simulated band structure of infinitely long 3,1,w-chGNRs 15

Supplementary Note 5 Extended SSH model to simulate the band structure of z, a, w-chGNRs 16

Supplementary Note 6 Tight Binding simulation of frontier and bands end states in finite chGNRs 18

Supplementary Note 7 Complementary scanning tunneling spectroscopy results of 3,1,8-chGNRs 19

Supplementary Note 8 Analysis of low-energy band structure of 3,1,6-chGNRs 20

Supplementary Note 9 Mean-field Hubbard simulations of finite chGNRs 22

Part I

Supplementary Methods

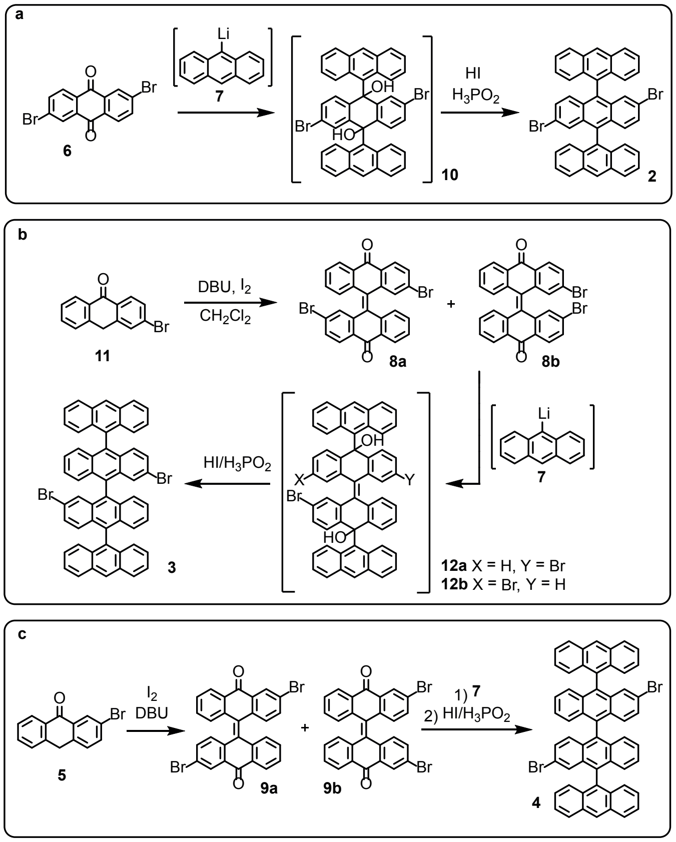

Supplementary Figure 1: Synthetic procedures for the preparation of (a) trisanthracene **2** (b) tetrakisanthracene **3** and (c) tetrakisanthracene **4**.

Synthesis routes of organic precursors Supplementary Figure **1** summarizes the synthetic procedures followed for the preparation of trisanthracene **2** and tetrakisanthracenes **3** and **4**. Bisanthracene **1**, dibromoanthraquinone **6**, and bromoanthrones **5** and **11** were prepared following reported procedures[1–3].

Synthesis of 2,6-dibromoanthracene-9,10-dione (6): Over a mixture of diaminoanthraquinone **13** (1.00 g, 4.20 mmol) and $CuBr_2$ (2.34 g, 10.5 mmol) in MeCN (50 mL), tBuONO (1.24 mL, 10.5 mmol) was added and the resulting mixture was heated at reflux for 16 h. After cooling to 0 C, aqueous HCl (20% wt, 10 mL) was added. The thus formed precipitate was filtered, affording **6** (1.46 g, 95%) as a brown solid.

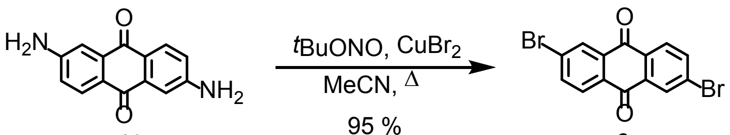

Supplementary Figure 2: Synthesis of 2,6-dibromoanthracene-9,10-dione (**6**).

^{1}H NMR (300 MHz, $CDCl_3$) δ: 8.43 (s, 2H), 8.17 (d, J = 8.3 Hz, 2H), 7.94 (d, J = 8.3 Hz, 2H) ppm.

Synthesis of 2',6'-dibromo-9,9':10',9''-teranthracene (2): Over a solution of organolithium **7** (30 mL, 1.37 mmol, 0.05 M inTHF), a suspension of anthraquinone 6 (200 mg, 0.55 mmol) in THF (10 mL) was added at room temperature (RT). The resulting mixture was stirred for 20 h at RT. Then, AcOH (1.0 mL) was added and volatiles were removed under reduced pressure. The residue (compound 10) was dissolved in AcOH (20 mL), and a mixture of H_3PO_2 (5.00 mL, 45.5. mmol, 50%) and HI (0.80 mL, 6.08 mmol, 57%) were added and the resulting mixture was heated at 80 C for 2 h. After cooling to RT, the mixture was diluted with CH_2Cl_2 (60 mL) and H_2O (60 mL), the phases were separated and the organic layer was washed with $NaHCO_3$ (saturated aqueous solution, 3x30 mL). The organic phase was dried over anhydrous Na_2SO_4, filtered and evaporated. The residue was purified by column chromatography (SiO_2; Hex:CH_2Cl_2 4:1 to 7:3), affording **2** (77 mg, 21%) as a yellow solid.

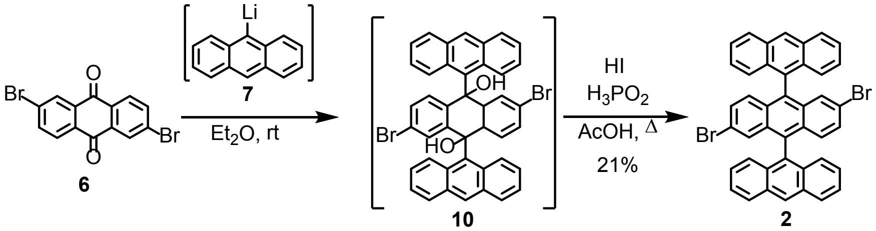

Supplementary Figure 3: Synthesis of 2',6'-dibromo-9,9':10',9''-teranthracene (**2**).

^{1}H NMR (363 K, 500 MHz, $C_2D_2Cl_4$) δ: 8.72 (s, 2H), 8.18 (d, J = 8.5 Hz, 4H), 7.50 (dd, J = 8.9, 5.9 Hz, 4H), 7.34 (d, J = 2.0 Hz, 2H), 7.32–7.26 (m, 4H), 7.21 (d, J = 8.7 Hz, 4H), 7.11 (dd, J = 9.3, 2.0 Hz, 2H), 7.01 (d, J = 9.3 Hz, 2H) ppm. ^{13}C NMR-DEPT (363 K, 125 MHz, $C_2D_2Cl_4$) δ: 134.2 (2C), 132.8 (4C), 131.9 (6C), 131.7 (2C), 130.7 (2C), 130.6 (2C), 130.2 (2CH), 129.4 (2CH), 129.1 (6CH), 128.3 (2CH), 126.8 (4CH), 126.6 (4CH), 125.8 (4CH) ppm. MS (EI) m/z (%): 688 (M+, 100), 522 (9), 344 (22), 261 (22), 255 (28). HRMS: $C_{42}H_{24}Br_2$; calculated: 686.0245, found: 686.0224.

Synthesis of bianthrones 8a and 8b: Over a solution of anthrone **11** (500 mg, 1.84 mmol) in CH_2Cl_2 (20 mL), DBU (0.97 mL, 6.50 mmol) was added and the mixture was stirred for 10 min.

Then, iodine (514 mg, 2.02 mmol) was added and the resulting solution was stirred in abstence of light for 24 h. Then, HCl (10%, 10 mL) and saturated aqueous solution of $Na_2S_2O_3$ (20 mL) were added, the organic layer was separated and aqueous phase was extracted with CH_2Cl_2 (2x20 mL). Combined organic extracts were dried over Na_2SO_4, filtered and evaporated under reduced pressure. The residue was purified by column chromatography (SiO_2; CH_2Cl_2), affording a mixture of isomers **8a** and **8b** (1:1, 302 mg, 61%) as an orange solid.

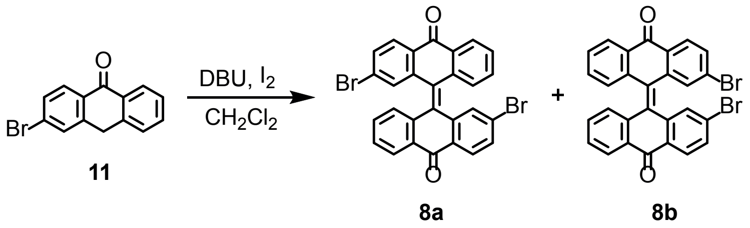

Supplementary Figure 4: Synthesis of bianthrones **8a** and **8b**.

MS (EI) m/z (%): 542 (M+, 100), 381 (13), 353 (16), 324 (33), 162 (50). HREM: $C_{28}H_{14}O_2Br_2$: calculated: 539.9361, found: 539.9368. Isomer 1: ^{1}H NMR (300 MHz, $CDCl_3$) δ: 8.14 (d, J = 8.0 Hz, 1H), 8.00 (d, J = 8.6 Hz, 1H), 7.62 (dd, J = 8.3, 1.9 Hz, 1H), 7.55–7.43 (m, 1H), 7.31–7.25 (m, 1H), 7.20 (d, J = 1.9 Hz, 1H), 7.03 (t, J = 7.1 Hz, 1H) ppm. Isomer 2: 1H NMR (300 MHz, $CDCl_3$) δ: 8.10 (d, J = 7.8 Hz, 1H), 7.97 (d, J = 9.3 Hz, 1H), 7.55 (dd, J = 8.3, 1.8 Hz, 1H), 7.50–7.37 (m, 1H), 7.23–7.13 (m, 1H), 7.15 (d, J = 1.8 Hz, 1H), 7.03 (t, J = 7.1 Hz, 1H) ppm.

Synthesis of 2”,3’-dibromo-9,9’:10’,9”:10”,9”’-quateranthracene (3): Over a solution of organolithium **7** (5.0 mL, 0.42 mmol, 0.08 M in Et_2O), a suspension of bianthrones **8a** and **8b** (50 mg, 0.09 mmol) was added in Et_2O (15 mL) at RT. The resulting mixture was stirred for 20 h at RT. Then, AcOH (1 mL) was added and volatiles were removed under reduced pressure. The residue (compounds **12a-b**) was redissolved in AcOH (10 mL), and a mixture of H_3PO_2 (1.00 mL, 9.10. mmol, 50%) and HI (0.50 mL, 3.80 mmol, 57%) were added and heated at 80 C for 2 h. After cooling to RT, the mixture was diluted with CH_2Cl_2 (50 mL) and H_2O (50 mL), the phases were separated and the organic layer was washed with $NaHCO_3$ (saturated solution, 3x20 mL). The organic phase was dried over anhydrous Na_2SO_4, filtered and evaporated. The residue was purified by column chromatography (SiO_2; Hex:CH_2Cl_2 4:1), affording **3** (32 mg, 40%) as a yellowish solid.
^{1}H NMR (300 MHz, CD_2Cl_2) δ: 8.84 (s, 2H), 8.28 (d, J = 8.5 Hz, 4H), 7.67 (d, J = 1.9 Hz, 2H), 7.61–7.53 (m, 4H), 7.44–7.18 (m, 20H) ppm. ^{13}C NMR-DEPT (75 MHz, CD_2Cl_2) δ: 135.9 (2C), 133.1 (4C), 132.9 (2C), 132.7 (2C), 132.4 (2C), 132.3 (8C), 130.5 (2C), 130.0 (2CH), 129.9 (2CH), 129.4 (4CH), 129.1 (2CH), 128.4 (2CH), 128.0 (2CH), 127.6 (2CH), 127.5 (2CH), 127.2 (2CH), 127.1 (2CH), 127.0 (4CH), 126.9 (2CH), 126.2 (2CH), 126.1 (2CH), 121.6 (2C)ppm. MS NALDI-TOF $C_{56}H_{32}Br_2$, calculated: 862.09, found: 861.79.

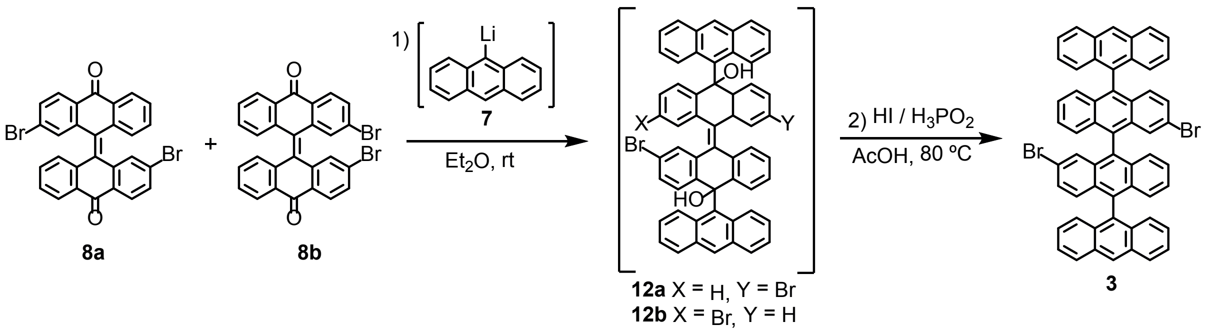

Supplementary Figure 5: Synthesis of 2”,3’-dibromo-9,9’:10’,9”:10”,9”’-quateranthracene (**3**)

Synthesis of bianthrones 9a and 9b: Over a solution of anthrone **5** (500 mg, 1.84 mmol) in CH_2Cl_2 (20 mL), DBU (0.97 mL, 6.50 mmol) was added and the mixture was stirred for 10 min. Then, iodine (514 mg, 2.02 mmol) was added and the resulting solution was stirred in abstence of light for 24 h. Then, HCl (10%, 10 mL) and saturated aqueous solution of $Na_2S_2O_3$ (20 mL) were added, the organic layer was separated and the aqueous phase was extracted with CH_2Cl_2 (2x20 mL). The combined organic phases were dried over Na_2SO_4, filtered and evaporated under reduced pressure. The crude product was purified by column chromatography (SiO_2; CH_2Cl_2), affording a mixture of isomers **9a** and **9b** (1:1, 271 mg, 55%) as an orange solid.

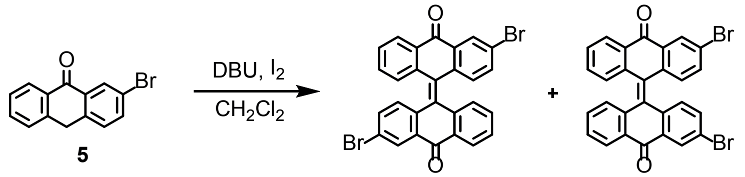

Supplementary Figure 6: Synthesis of bianthrones **9a** and **9b**

MS (EI) m/z (%): 542 (M+, 100), 381 (32), 324 (34), 162 (48). HRMS: $C_{28}H_{14}O_2Br_2$: calculated: 539.9361, found: 539.9364. Isomer 1: ^{1}H NMR (300 MHz, $CDCl_3$) δ: 8.23 (d, J = 2.4 Hz, 1H), 8.09 (ddd, J = 7.7, 1.3, 0.5 Hz, 1H), 7.51–7.39 (m, 1H), 7.34 (dd, J = 8.3, 2.1 Hz, 1H), 7.24 (ddd, J = 8.0, 7.4, 1.4 Hz, 1H), 7.08 (d, J = 8.1 Hz, 1H), 6.97 (d, J = 8.4 Hz, 1H) ppm. Isomer 2: ^{1}H NMR (300 MHz, $CDCl_3$) δ: 8.22 (d, J = 2.2 Hz, 1H), 8.09 (ddd, J = 7.6, 1.3, 0.5 Hz, 1H), 7.45 –7.39 (m, 1H), 7.28 (ddd, J = 8.0, 7.4, 1.4 Hz, 1H) , 7.17 (ddd, J = 8.0, 7.4, 1.4 Hz, 1H), 7.04 (dd, J = 8.0, 0.7 Hz, 1H), 6.91 (d, J = 8.3 Hz, 1H) ppm

Synthesis of 2’,3”-dibromo-9,9’:10’,9”:10”,9”’-quateranthracene (4): Over a solution of organolithium **7** (2.0 mL, 0.20 mmol, 0.10 M in Et_2O) a suspension of bianthrones **9a** and **9b** (50 mg, 0.09 mmol) was added in Et_2O (4 mL) at RT. The resulting mixture was stirred for 20 h at RT. Then, AcOH (1 mL) was added and volatiles were removed under reduced pressure. The residue (compounds **14a** and **14b**) was redissolved in AcOH (10 mL), a mixture of H_3PO_2 (1.00 mL, 9.10.

mmol, 50%) and HI (0.50 mL, 3.80 mmol, 57%) were added and the resulting mixture was heated at 80 C for 2 h. After cooling to RT, the mixture was diluted with CH_2Cl_2 (50 mL) and H_2O (50 mL), the phases were separated and the organic layer was washed with $NaHCO_3$ (saturated solution, 3x20 mL). The organic phase was dried over anhydrous Na_2SO_4, filtered and evaporated. The residue was purified by column chromatography (SiO_2; Hex:CH_2Cl_2 4:1 to 6:4), affording **4** (26 mg, 33%) as a yellowish solid.

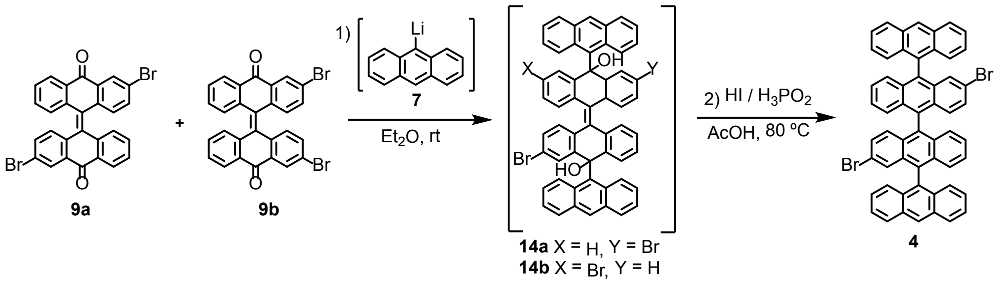

Supplementary Figure 7: Synthesis of compound **4**

^{1}H NMR (353K, 500 MHz, $C_2D_2Cl_4$) δ: 8.74 (s, 2H), 8.20 (d, J = 8.6 Hz, 4H), 7.50 (d, J = 7.3 Hz, 4H), 7.44–7.36 (m, 4H), 7.34–7.10 (m, 18H) ppm. ^{13}C NMR-DEPT (353 K, 100 MHz, $C_2D_2Cl_4$) δ: 134.1 (2CH), 132.0 (4CH), 130.1 (2CH), 129.1 (4CH), 128.2 (2CH), 127.6 (2CH), 127.3 (2CH), 126.9 (2CH), 126.8 (4CH), 126.7 (4CH), 125.8 (4CH) ppm. MS (EI) m/z (%): 864 (M+, 100), 432 (28). HRMS: $C_{56}H_{32}Br_2$; calculated: 862.0871, found: 862.0864.

^{1}H and ^{13}C NMR spectra

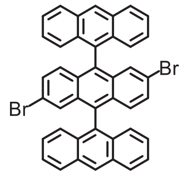

2

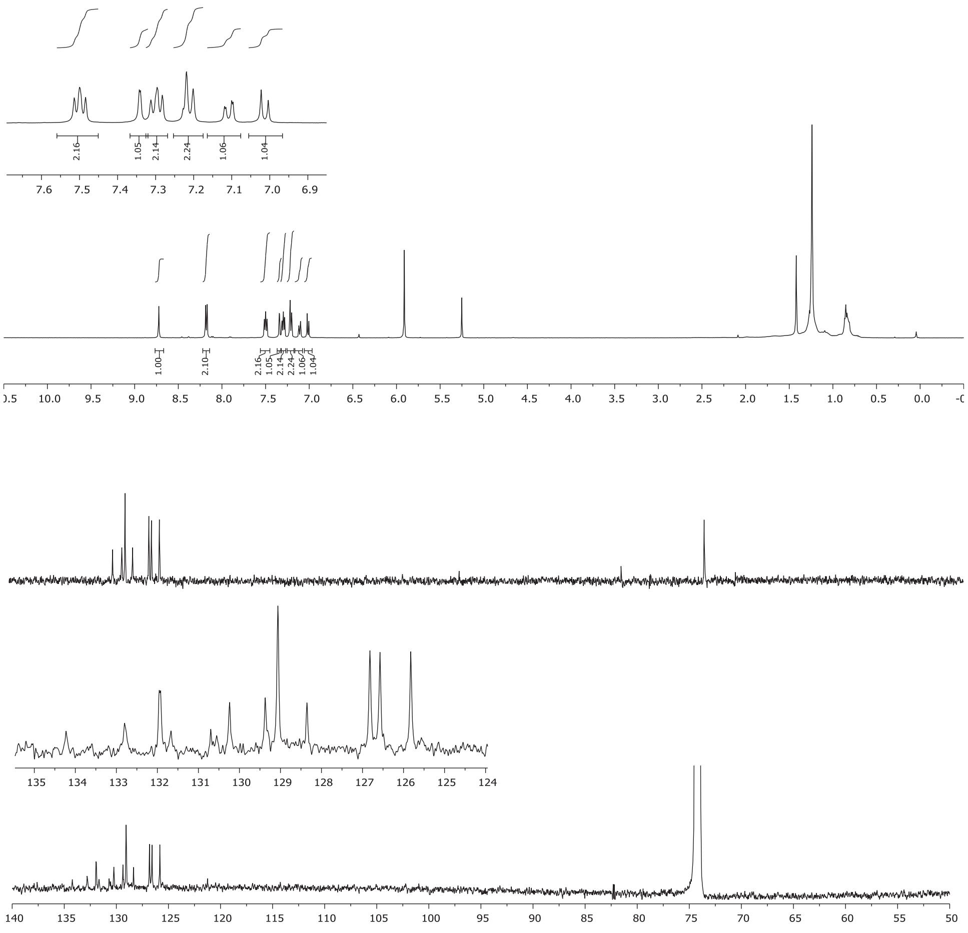

Supplementary Figure 8: ^{1}H and ^{13}C NMR spectra of 2’,6’-dibromo-9,9’:10’,9’’-teranthracene (**2**)

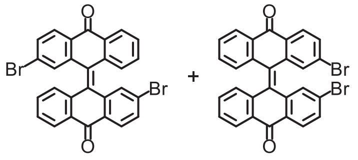

8a **8b**

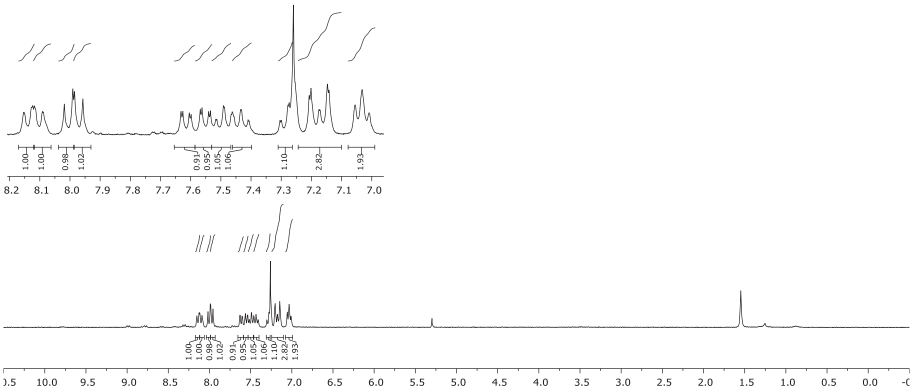

Supplementary Figure 9: ^{1}H and ^{13}C NMR spectra of bianthrones **8a** and **8b**

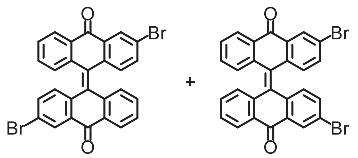

9a **9b**

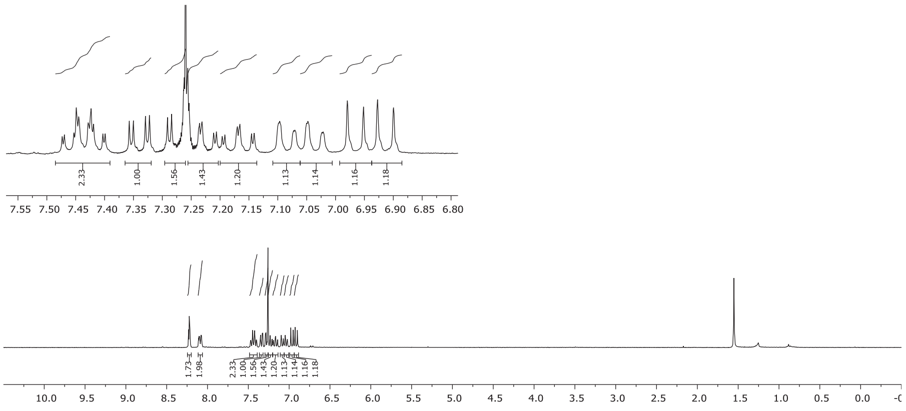

Supplementary Figure 10: ^{1}H and ^{13}C NMR spectra of bianthrones **9a** and **9b**

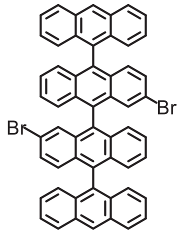

3

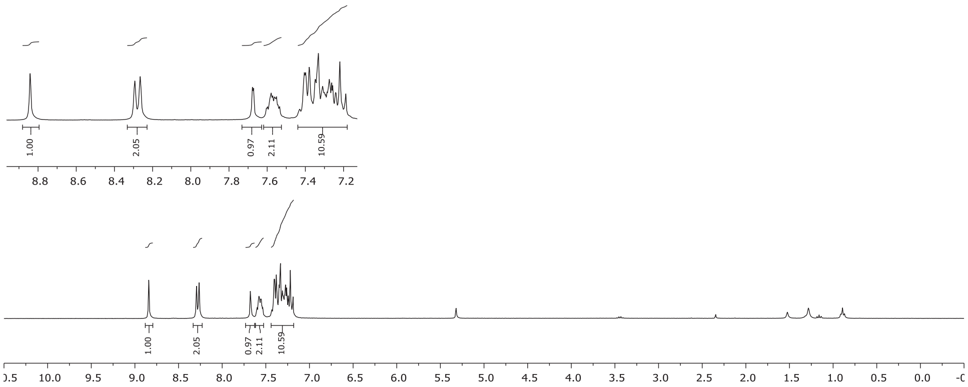

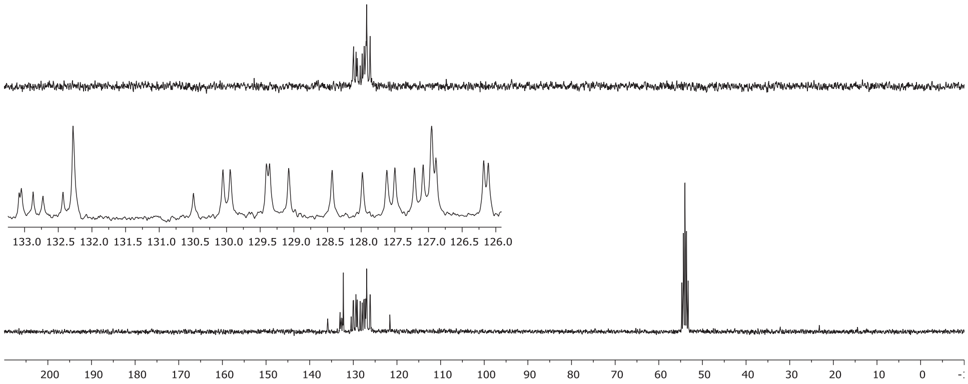

Supplementary Figure 11: ^{1}H and ^{13}C NMR spectra of 2'',3'-dibromo-9,9':10',9'':10'',9'''-quateranthracene (**3**)

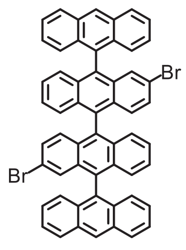

4

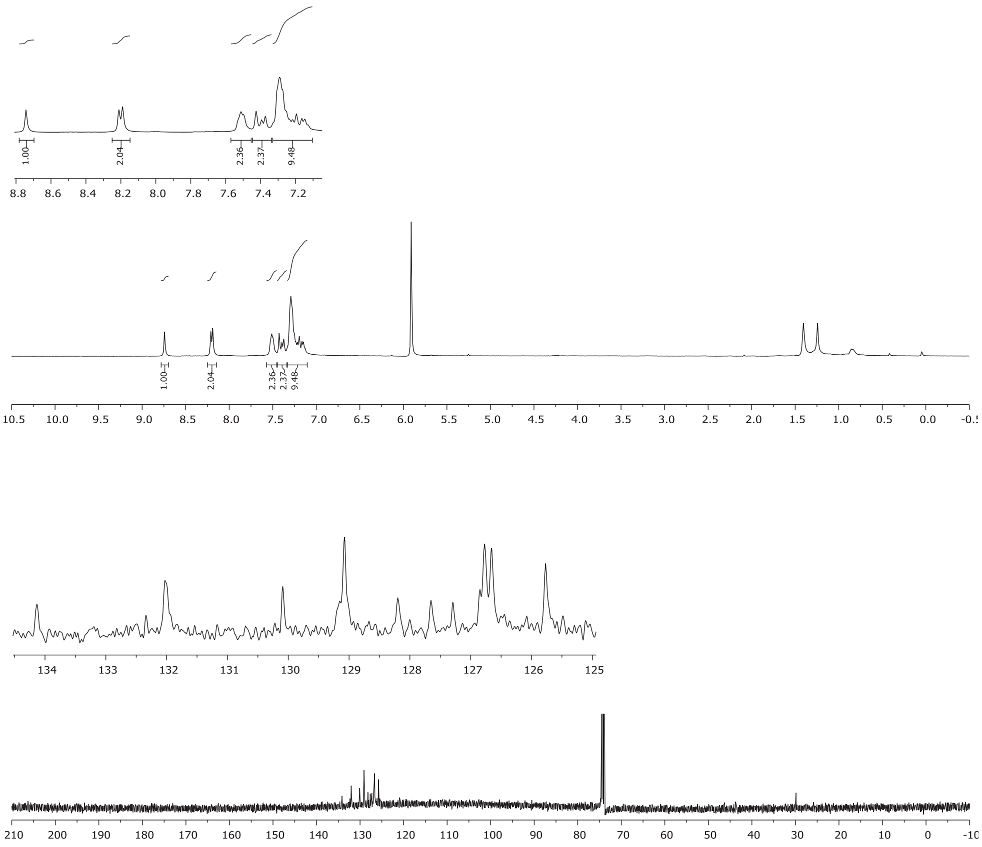

Supplementary Figure 12: ^{1}H and ^{13}C NMR spectra of 2’,3”-dibromo-9,9’:10’,9”:10”,9”’-quateranthracene (**4**)

Part II

Supplementary Notes

Supplementary Note 1 Structure of the family of chiral GNRs studied here

Chiral GNRs are generally defined by their chiral vector (n, m), where n and m are indexes of the graphene unit vectors. In general, it is usual to consider $n > m$. This label is not specific with the structure of the edges, many of which can accommodate to follow the GNR chiral vector. In this study we consider a subfamily of chiral GNRs constructed by laterally joining rectangular pieces of graphene of width z zigzag unit cells and length amounting to w carbon atoms. The rectangular unit blocks are connected one to another through specific bonds, labelled a in Supplementary Fig. 13a. The a label also indicates the shift between neighbour blocks, $a - 1/2$, in units of armchair unit cells. Hence, this family comprehends a set of (n, m) chGNRs with an specific edge structure formed by alternating z zigzag units and $a - 1/2$ perpendicular armchair sites (see Supplementary Fig. 13b). In the manuscript we labelled this family as z, a, w-chGNR. In this way, the three parameters z, a, w determine width, edge structure, and the chiral vector $(m, n) = (z+1-a, 2a-1)$ or chiral angle $\phi = \arctan\sqrt{\frac{3}{4}\frac{2a-1}{z+1/2}}$.

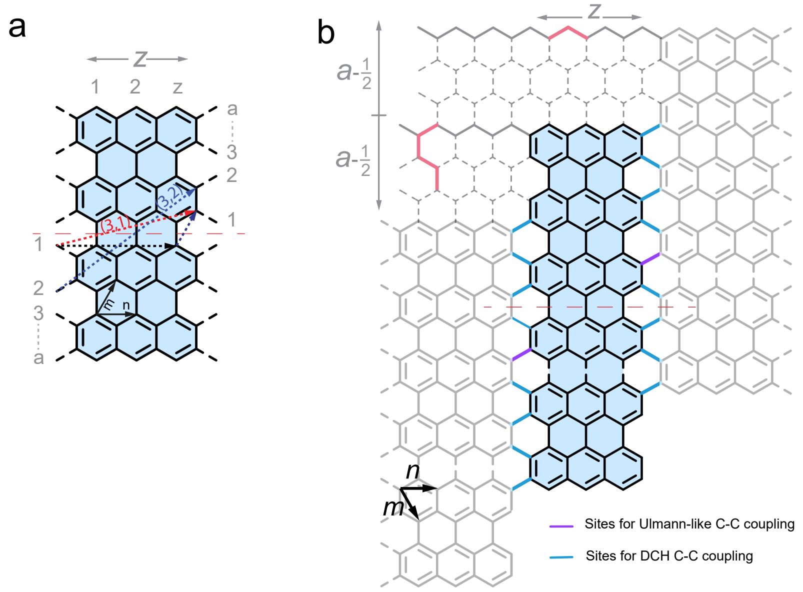

Supplementary Figure 13: **a**, Schema of a basic unit block used in the mansucript for the $z = 3$, $w = 8$ chiral GNRs **b**, Diagram of the connection pattern of two $z = 3$, $w = 12$ unit blocks connected along the $a = 2$ sites. We show in purple the a sites, used for inducing Ullmann coupling on a metal substrate. Blue bonds are C-C bonds created in the second cyclodehydrogenation (CDH) step, at higher temperatures. Pink lines mark length of zigzag and armchair unit lengths.

Supplementary Note 2 Complementary Tight Binding simulations

Results with first-nearest neighbor tight binding model: The results presented in the main manuscript have been computed with up to third-nearest neighbor (3NN) interactions. For completeness, we have compared the band structures, gaps and SPT phase with simulations with a first-nearest neighbor (1NN) model (i.e. with $t_1 = 2.7$ eV and $t_2 = t_3 = 0$), which is typically employed to study graphene nanostructures [4]. As an example, we show in Supplementary Fig. 14 (a-c) the computed the band structures for the 3,1,w-chGNRs ($w = 4, 6, 8$) and in Supplementary Fig. 14(d) the complete phase diagram with this particular choice of parameters. The main claim of our manuscript regarding a topological phase transition in narrow chGNRs is also observed with this model.

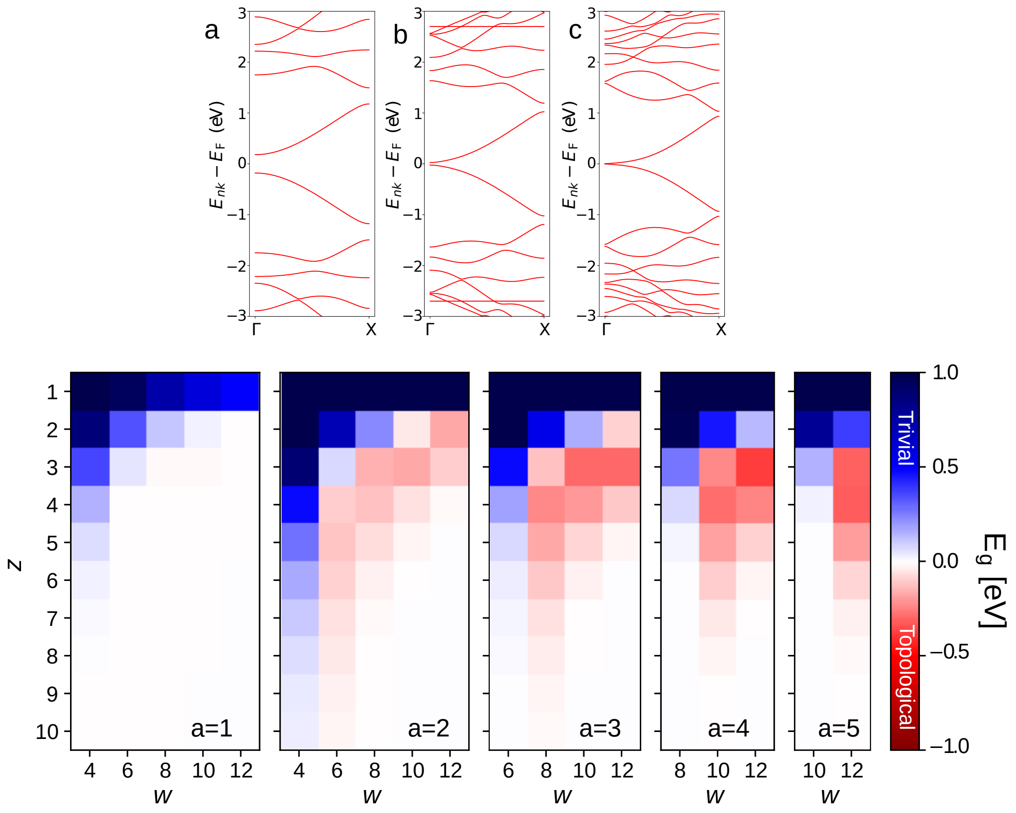

Supplementary Figure 14: (a,b,c) Band structures obtained with the 1NN model for the (3,1,4), (3,1,6) and (3,1,8) chGNRs, respectively. (d) Color plots of the energy gap value multiplied by $(-1)^{\mathbb{Z}_2}$ for the family of z, a, w-chGNRs obtained with the 1NN model.

Band structures and VB wave functions of 3,2,8-chGNRs: The valence band (VB) wave function of the 3,1,w-chiral ribbons has inversion symmetry. As we reported in the main manuscript, a non-trivial SPT phase transition is reflected in a wave function with different parity at the center of the Brillouin center with respect to the boundary. As we show in Supplementary Fig. 15, this is also the case for 3,2,8-chGNR. The VB wave function is even at $k = 0$ and odd at $k = \pi$, meaning that electrons accumulate a phase as they disperse along this band. This coincides with the Zak phase of π obtained both for this band, as well as for the global band structure.

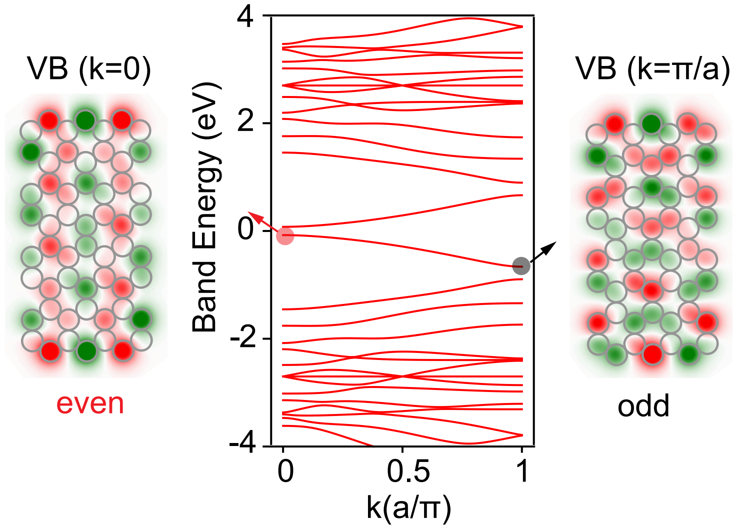

Supplementary Figure 15: **Band structure of infinite 3,2,8-chGNRs:** Middle panel, tight binding simulated band structures of infinitely long 3.2.8-chGNRs, with the wave function of the VB at Γ ($k = 0$) and X ($k = \pi/a$) shown in the left and right panels respectively. The change in inversion symmetry of the wave function shows that for this unit cell the ribbons gains a Zak phase of π as it disperses along the Brillouin zone and, accordingly, it is in a topological insulating state.

Supplementary Note 3 Determination of the topological phases of chiral GNRs

For one dimensional (1D) gapped systems the surface charge at the end of such an insulator is directly related to the Zak phase γ [5]. For inversion/mirror symmetric Bloch Hamiltonians, this phase is quantized to values 0 or π (mod 2π) [6,7], which allows for a topological classification for these 1D systems. The topological invariant of an inversion symmetric 1D unit cell, $\mathbb{Z}_2$, hence can be defined by a simple relation to the Zak phase $(-1)^{\mathbb{Z}_2} = e^{i\gamma}$, that can only have values of 0 or 1.

In this section we apply this model to establish a topological classification of graphene nanoribbons according to three different properties: i) their Zak phase and its related band parity $\hat{P}$, ii) their band gap, and iii) the apparition of end states.

Calculation of the Zak phase for chiral GNRs: We start by defining the Hamiltonian of the unit cell of our 1D chiral ribbons for a k point, H_k, and the Bloch wave functions resulting from its diagonalization ψ_{nk},

$$H_k\psi_{nk} = \varepsilon_{nk}\psi_{nk}, \qquad \psi_{nk} = e^{-ik}u_{nk}, \tag{1}$$

where n is the band index and u_{nk} satisfies the periodic boundary conditions over the unit cell. For a 1D system, the Zak phase is the integration of the Berry connection along an open contour over the first Brillouin zone [5],

$$\gamma = \sum_n \gamma_n = \sum_n \int_{-\pi/d}^{\pi/d} dk \, \langle u_{nk}|i\nabla_k|u_{nk}\rangle \tag{2}$$

where d is the size of the unit cell and the summation runs over the occupied bands. Note that there is some arbitrariness in identifying the nth band at crossing bands points. However, the total phase is gauge invariant and remains unaffected by such arbitrariness if the system is an insulator (the occupied bands do not cross the empty ones at any point of the path) [8]. For practical purposes, we calculate this integral by discretizing the open contour in $N+1$ points, as implemented in the Python package SISL [9],

$$\gamma \rightarrow -\text{Im}\log\det\prod_{i}^{N-1} \langle u_{nk_i}|\, u_{n'k_{i+1}}\rangle \tag{3}$$

The periodic boundary conditions impose that $u_{nk+G} = e^{-iGr}u_{nk}$ in the chosen gauge, with G being the reciprocal lattice vector. In the discretized picture of Eq. (3), this relation gives us the connection between the first and last points of the contour, $\langle u_{nk_{M-1}} \left| e^{-iGr} \right| u_{n'k_0}\rangle$.

This $\mathbb{Z}_2$ invariant is also related to the parity of the Bloch wave functions at the time-reversal-invariant (TRI) k-points ($\Gamma_i \in \{0, \pi/a\}$ for 1D systems) in the Brillouin Zone by [6],

$$(-1)^{\mathbb{Z}_2} = \prod_n \chi_n(0)\chi_n(\pi/2) \tag{4}$$

where $\chi_n(\Gamma_i) = \langle\psi_{n\Gamma_i}|\hat{P}|\psi_{n\Gamma_i}\rangle$ is the eigenvalue of the parity operator $\hat{P}$ for the Bloch orbital $\psi_{n\Gamma_i}$ at the TRI points Γ_i, and the product runs over the occupied bands.

Supplementary Note 4 DFT simulated band structure of infinitely long 3,1,w-chGNRs

In the manuscript we discuss the band structure of infinitely long 3,1,w-chGNRs based on TB simulations. To complement the TB simulations, we also performed DFT simulations of band structures of infinitely long 3,1,w-chGNRs, as shown in Supplementary Figure 16. DFT simulations find clear gap closing from 3,1,4-chGNRs to 3,1,6-chGNRs and 3,1,8-chGNRs. Furthermore, the gap of 3,1,6-chGNRs is slightly smaller than 3,1,8-chGNRs, which matches the trend from TB simulations.

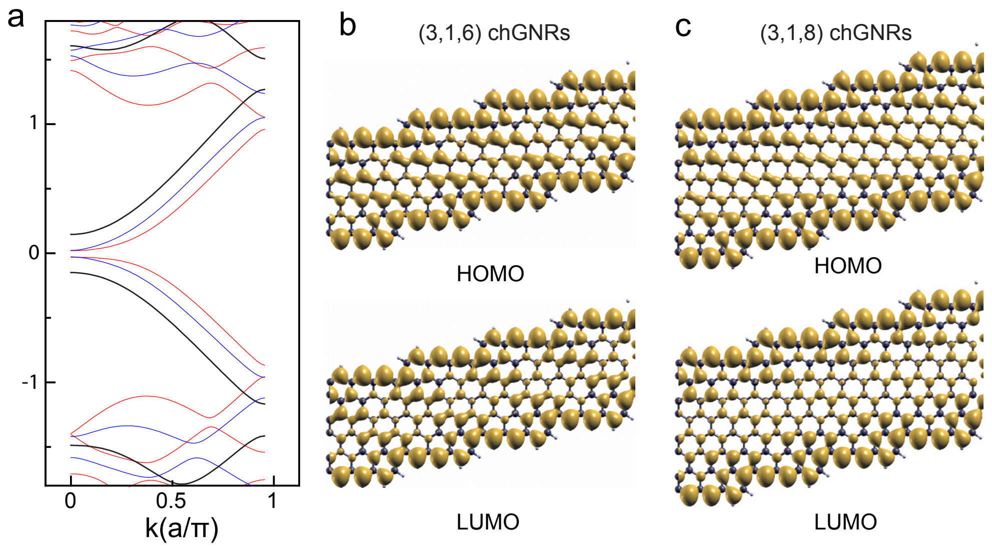

Supplementary Figure 16: DFT simulated band structure LDOS of (3,1) chGNRs. (a) Black, blue and red curves are DFT simulated band structures of infinitely long (3,1,4), (3,1,6) and (3,1,8) chGNRs. (b,c) DFT simulated LDOS of VB and CB of (3,1,6) and (3,1,8) chGNRs.

Supplementary Note 5 Extended SSH model to simulate the band structure of z, a, w-chGNRs

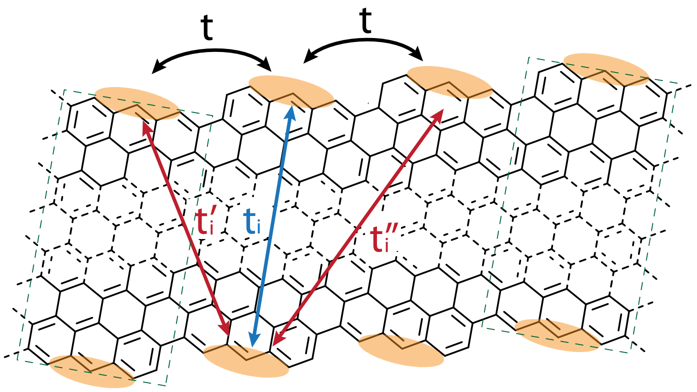

Supplementary Figure 17: Four effective hopping parameters used in a modified SSH toy model to simulate the SPT phase transition.

We use the toy model depicted in Supplementary Fig. 17 with four effective parameters (extended SSH chain) used to fit the low-energy bands of the chGNRs and obtain the expected topological invariant justified up to a geometrical level. In this model we consider a hopping parameter t for electrons along the edges (representing the coupling between zigzag states spaced by the armchair segments at the edge), and three *width-dependent* hopping parameters between zigzag states at opposing edges of the ribbon. One of them, (t_i), represents the coupling of intra-cell states, and the other two ($t_i^{'}, t_i^{''}$) between opposing zigzag states in neighbouring cells. The model Hammiltonian is:

$$H = H_{00} + H_{10}e^{ik} + H_{-10}e^{-ik} = \begin{pmatrix} 0 & t_i \\ t_i & 0 \end{pmatrix} + \begin{pmatrix} t & t_i^{'} \\ t_i^{''} & t \end{pmatrix} e^{ik} + \begin{pmatrix} t & t_i^{''} \\ t_i^{'} & t \end{pmatrix} e^{-ik} \tag{5}$$

This model has analytical solutions when solving the eigenvalue problem, which are the valence (E_-) and conduction (E_+) bands,

$$E_{\mp}(k) = t\cos(k) \mp \sqrt{t_i^{'2} + t_i^2 + t_i^{''2} + 2t_i(t_i^{'} + t_i^{''})\cos(k) + 2t_i^{'}t_i^{''}\cos(2k)} \tag{6}$$

The parameter t introduces particle-hole asymmetry in Eq. (6). Even in the case of 3NN model, this asymmetry is quite insignificant (especially for the low energy bands). For this reason the effective coupling of the orbitals that lie along the same chGNR edge is practically zero.

In Supplementary Figure 18 we plot the band structures for each chGNR in black solid lines, and in red we include the resulting bands fitted with the extended SSH model of above. In Table 1, we collect the numerical values for the parameters t, t_i, $t_i^{'}$ and $t_i^{''}$ obtained from the fits for the low energy bands for each z, a, w-chGNRs. From Eq. (6) one easily deduces a gap closing when $-t_i = (t_i^{'} + t_i^{''})$. At this point, the topological phase transition takes place. This is precisely what we observe in Table 1 and Supplementary Fig. 18, where the topological transition occurs for

(n,m,w)	t	t_i	$t_i^{'}$	$t_i^{''}$	Z_2	E_g
$(3,1,4)$	0.001	-0.528	0.212	0.212	0	0.210
$(3,1,6)$	$1.00 \cdot 10^{-5}$	-0.430	0.213	0.212	0	0.010
$(3,1,8)$	$1.62 \cdot 10^{-5}$	-0.392	0.213	0.193	1	0.011
$(3,1,10)$	0.000	-0.388	0.206	0.187	1	0.006
$(3,2,4)$	0.001	-0.381	0.000	0.000	0	0.761
$(3,2,6)$	0.000	-0.263	0.178	0.068	0	0.034
$(3,2,8)$	0.000	-0.199	0.244	0.063	1	0.216
$(3,2,10)$	0.000	-0.183	0.223	0.067	1	0.207

Table 1: Table of the parameters that better fit the low energy bands of each (n,m,w) (obtained with 3NN) and the resulting Z_2 invariant of the toy model. All values are given in eV.

widths around the $(3,1,6)$ and $(3,2,6)$ systems. The band gap shown in column 7 in Table 1 is obtained as a numerical search of the minimum value of the energy distance between the valence and conduction bands (direct gap), which for most of the cases does not occur at Γ.

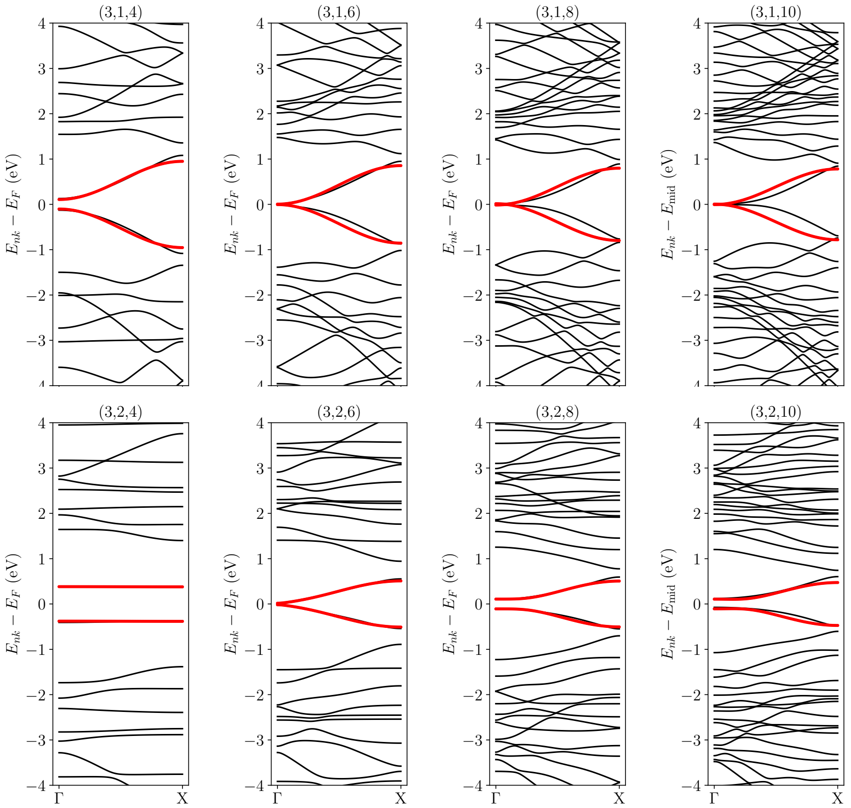

Supplementary Figure 18: Band structure of the n,m,w-chGNRs (black solid lines) obtained with the 3NN model and the bands from the SSH-like chain (red dots) with the fitting parameters corresponding to Table 1 for each particular chGNR.

Supplementary Note 6 Tight Binding simulation of frontier and bands end states in finite chGNRs

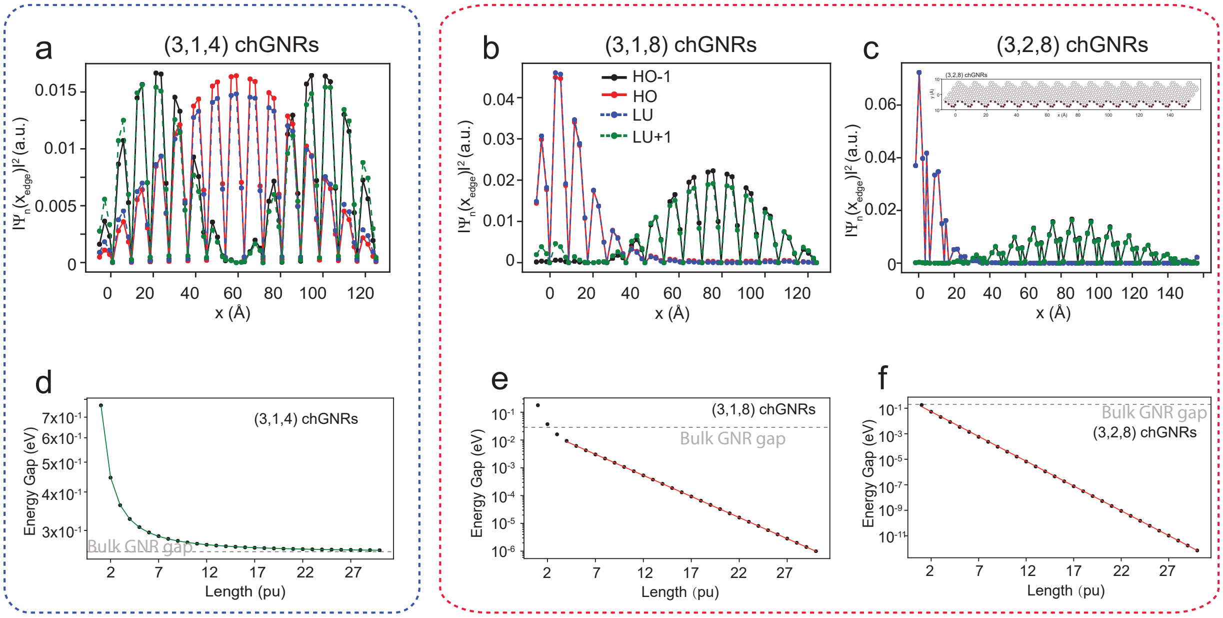

Supplementary Figure 19: **TB simulations of finite chGNRs: a-c** Wave function amplitude along the edges for the Highest Occupied (HO) and Lowest Unoccupied (LU) states of 15 precursor units long 3,1,4-, 3,1,8-, and 3,2,8-chGNRs (color labels indicated in **b**). Inset in panel **c** indicates edge positions where the amplitude is represented. **d-g** Evolution of HO-LU energy difference vs. chGNR length. In grey we indicate the energy gap of the infinite ribbon (VB-CB). In **d**, green curve plots the fitting function: $f(L) = e^{-aL}$. In **e,f** red plot are fitting function $f(L) = L^{-b}$. L stands for the length of the GNRs and a,b are the fitting parameters.

In Supplementary Figure 19**a-c** we compare the spatial distribution along the chGNR edge of the wave function amplitude of frontier states for the trivial 3,1,4-chGNR and for the topological 3,1,8-chGNR and 3,2,8-chGNR. The HO and LU states of the trivial 3,1,4-chGNR are distributed along the ribbon with maximum amplitude at the center, while HO-1 and LU+1 states show nodal plane in the middle of the ribbon. This distribution is caused by confinement into quantum well states due to the finite length of the ribbon, confirming that frontier states are VB and CB onset of the bulk ribbon, and the absence of intra-gap states.

In contrast, frontier states of the topological insulating 3,1,8-, and 3,2,8-chGNRs are SPT boundary states, localized at the chGNRs ends and split due to their mutual interaction (as shown in Supplementary Fig. 19**e-f**). The end state in **b**, extends for 4 PUs due to the smaller band gap of the 3,1,8-chGNR. In contrast, SPT end states in 3,2,8-chGNR are more localized in agreement with the larger band gap of this ribbon. For both topological ribbons, the next lower/higher states (HO-1 and LU+1) are distributed as the first quantum well state, being these the bulk edge states. These define the band gap of the insulating phase.

Supplementary Figs. 19**d-f** shows the evolution of the energy difference between frontier states with the length. For the trivial 3,1,4-chGNR, the energy gap closes with increasing length, and approaches the bulk energy gap of the infinite ribbon exponentially. This confirm that the HO and LU states correspond to GNR bulk bands, and the absence of SPT boundary states in this system. For the topological ribbons, the HO-LU gap closes as an inverse power law, and enters inside the bulk gap of an infinite ribbon for very short ribbons. This corroborates that this gap represents the small interaction between SPT end states.

Supplementary Note 7 Complementary scanning tunneling spectroscopy results of 3,1,8-chGNRs

Spectral evolution along the edge Due to the small band gap of 3,1,8-chGNRs, SPT end states extends along three precursor units (PU), decaying towards the interior of the edge band. Supplementary Figure 20a shows a set of spectra measured along the edge, at the positions indicated in Supplementary Supplementary Fig. 20b. The SPT boundary state is centered slightly above zero bias, and extends for about three precursor units. Beyond these, the valence and conduction band onsets emerge and a gapped feature dominate the. Note that the SPT state is only observed in one termini per edge.

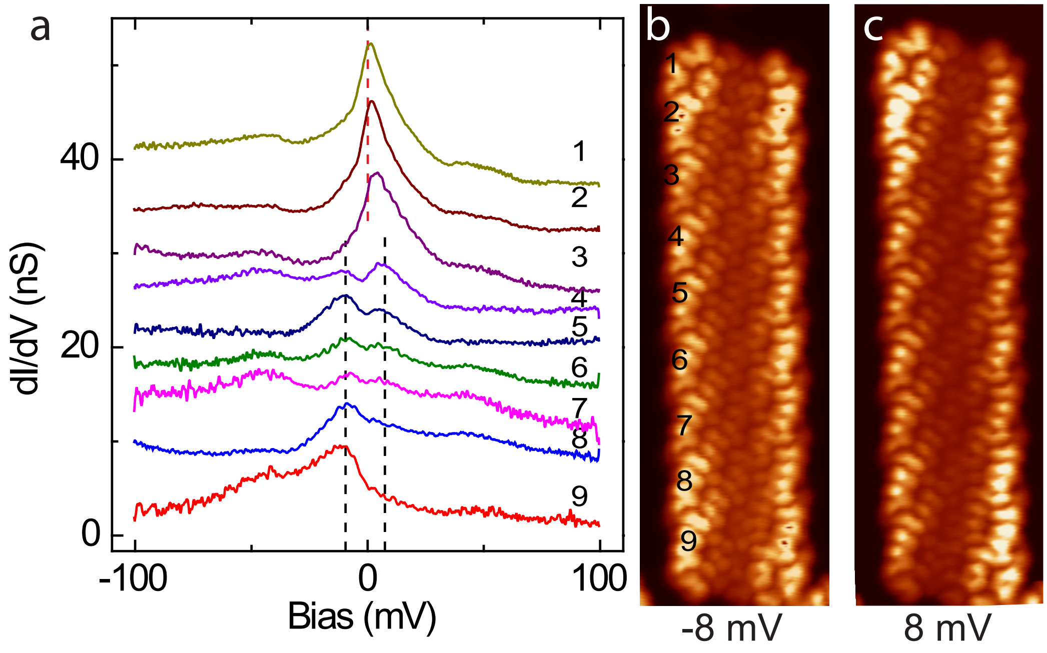

Supplementary Figure 20: **a** dI/dV spectra taken along an edge of the (3,1,8) chGNRs shown in **b**. **b,c** Constant height dI/dV maps measured at the indicated bias values. The maps were measured with a STP tip functionalized with a CO molecule.

Supplementary Note 8 Analysis of low-energy band structure of 3,1,6-chGNRs

We present here a set of data on 3,1,6-chGNRs described in the main text, which evidences that low-bias structure in spectra corresponds to the onset of frontier bands and the absence of topological end states at the termini. These results agree with the trivial character of this chGNR.

Identification of VB and CB: Supplementary Fig. 21 we show dI/dV measurements taken on a 3,1,6-chGNRs with 6 precursor units (pu). In Supplementary Fig. 21a, detailed narrow-range dI/dV spectra show three peaks at around -56 mV, 4 mV and 74 mV, which are distributed all along the ribbon. The LDOS at these energies are demonstrated by constant height dI/dV maps at the corresponding energies in Supplementary Fig. 21b-d). By comparing the LDOS with DFT simulated wave functions(Supplementary Fig. 21**e**), we attribute the states at 4 mV and 74 mV to the valence band (VB) and conduction band (CB) of the free ribbon[10]. The fact that VB appears at positive bias is due the electron affinity of the Au substrate [11–13]. A we show bellow, the peak at -56 mV is identified as the quantum-well state of the VB edge estates due to confinement by the finite length[10, 14].

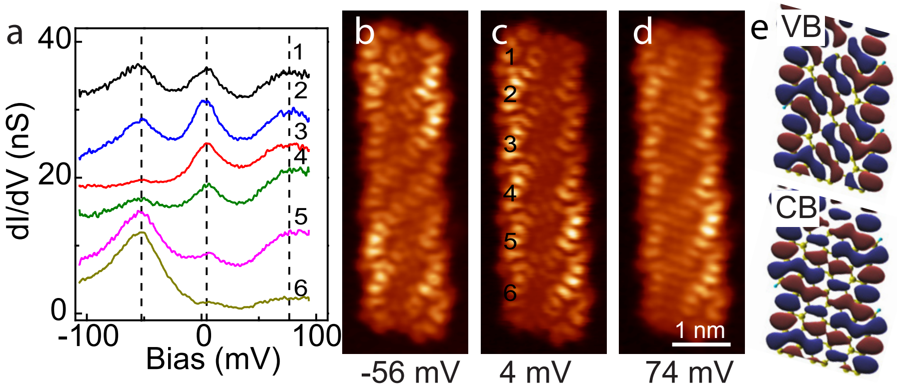

Supplementary Figure 21: **Edge states of (3,1,6) chGNRs.** **a**, dI/dV spectra taken on (3,1,6) chGNRs. The locations of the spectra are noted on image **c** with numbers. Gray dashed lines indicate the three peaks around Fermi level. **b-d**, dI/dV maps recorded at the energies of peaks on spectra in **a**. The bias values are noted on the maps. **e**, DFT simulations of the wave function for states at the onset of valence and conduction bands (at the Γ point). Red and blue colors represent isosurfaces of positive and negative wave function amplitudes, for an isovalue of 0.015 Å$^{-3/2}$.

Quantum well states in the edge bands: In Supplementary Fig. 22 we show detailed narrow-range dI/dV spectra taken on a longer 3,1,6-chGNR, with 16 precursor units (pu). The spectrum shows three peak features, as for the shorter ribbon in Supplementary Fig. 22a. Here, however, the peaks are at -25 mV, 30 mV and 70 mV. Supplementary Figure 22b shows constant current dI/dV maps at selected peak bias values. From the dI/dV structure along the ribbon, the two peaks at positive bias are identified as VB and CV onsets. The maps also reveal an additional energy-dependent LDOS modulation along the ribbon axis due to quantum confinement. For example, Supplementary Fig. 22d,e shows a dI/dV spectral map along the edge, and its first Fourier Transform. The two peaks at +30 and -25 appear there as energy discursive bands towards

negative bias values, thus confirming the VB-origin of both peak features in the spectra [10]. This quantum-well states in the edge bands of 3.1.6-chGNRs is reproduced by our TB simulated DOS shown in Supplementary Fig. 22f for a 10 PU ribbon.

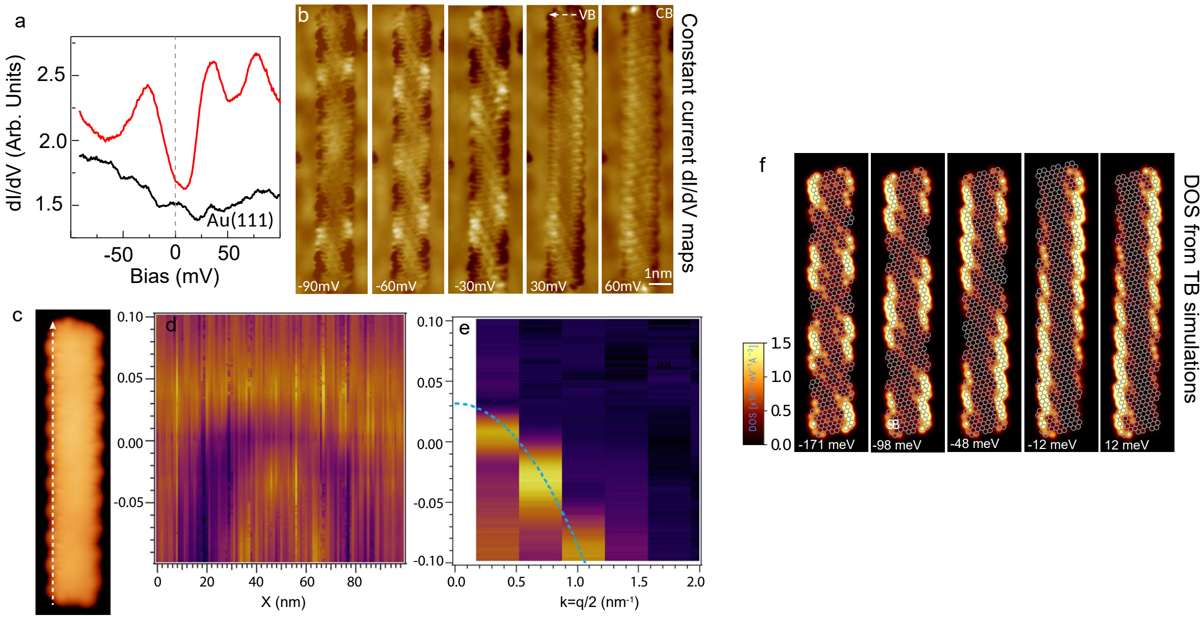

Supplementary Figure 22: **Size-confinement effects in the band structure of 3,2,6-chGNR a**, Differential conductance spectrum of a 3,1,6-chGNR with 10 precursor units (PUs, shown in **c**), and **b**, constant current dI/dV maps at the indicated bias. **c**, STM image ($V = 1$ V, $I = 45$ pA) of the studied (3,1,6) chGNRs with 10 PUs. **d**, 100 equidistant dI/dV point spectra taken along dashed line in **c**. (open-feedback: $V = 100$ mV, $I_t = 0.5$ nA; modulation: $V_{\mathrm{rms}} = 3$ mV) **e**, Line-by-line Fourier transform from equidistant spectra in **d**. The parabolic fit (blue dashed line) highlights the band dispersion. **f**, TB simulations of the DOS of a free 10 PU 3,1,6-chGNR at the indicated energy values, corresponding to discrete states of their band structure.

Trivial semiconductor character of 3.1.6-chGNR: In both figures of this note, the VB-CB band structure shows no in-gap state, neither characteristic signal enhancement, at the termini. Instead, the ribbon behaves as a trivial semiconductor.

Supplementary Note 9   Mean-field Hubbard simulations of finite chGNRs

In this section we analyze the effect of the electron-electron interactions in the (n, m, w)-chGNRs within the mean-field Hubbard (MFH) model, which has proven to describe sp^2 carbon systems with sufficient accuracy [4,15–19].

We performed calculations for finite molecules composed by a repetition of 9 blocks of the unit cell for each chGNR. In particular we study the projected density of states (PDOS) of (n, m, w)-chGNRs for different values of U. For these systems we compute the PDOS ($\mathrm{PDOS} = \mathrm{PDOS}_\uparrow + \mathrm{PDOS}_\downarrow$) at the end sites as a function of the electron energy for different U values for both the neutral molecule (N electrons) in Supplementary Fig. 23, and for the charged molecule with $N - 1$ ($N - 2$) electrons in Supplementary Fig. 29 (Supplementary Fig. 35). We also plot the real space distribution of the PDOS at different energies corresponding to the HOMO and LUMO in Supplementary Figs. 25-28 for the neutral system and in Supplementary Figs. 31-34 (Supplementary Figs. 37-40) for the charged system with one (two) electrons removed. For the cases with $Q_\uparrow = Q_\downarrow$ (neutral and with two electrons removed), as there is inversion symmetry, these energies are the same for both spin components. For the charged case with one electron removed there is no such symmetry.

From the MFH calculations we conclude that the wide band gap system (3,1,4)-cGNR remains essentially unpolarized, even for U up to 3 eV (neutral or charged cases). However, for this case, by comparing to the experimental dI/dV measurements we conclude that the (3,1,4)-chGNR does not donate electrons to the substrate since the experimental gap is significantly more similar to the simulated neutral system rather than the charged ones. On the other hand, the narrow gap systems [(3,1,6), (3,1,8), (3,2,8)] in the *neutral* state display spin polarization and opening of a correlation gap even at weak U. The spatial features of the end states survive up to $U \sim 2$ eV, but the correlation gap is not consistent with the experimental zero-energy peaks. The systems [(3,1,6), (3,1,8), (3,2,8)] in the *charged* state (both with one or two electrons removed) remain with end state features near zero energy for U up to the range 1–2.5 eV, quite consistent with the experimental observations.

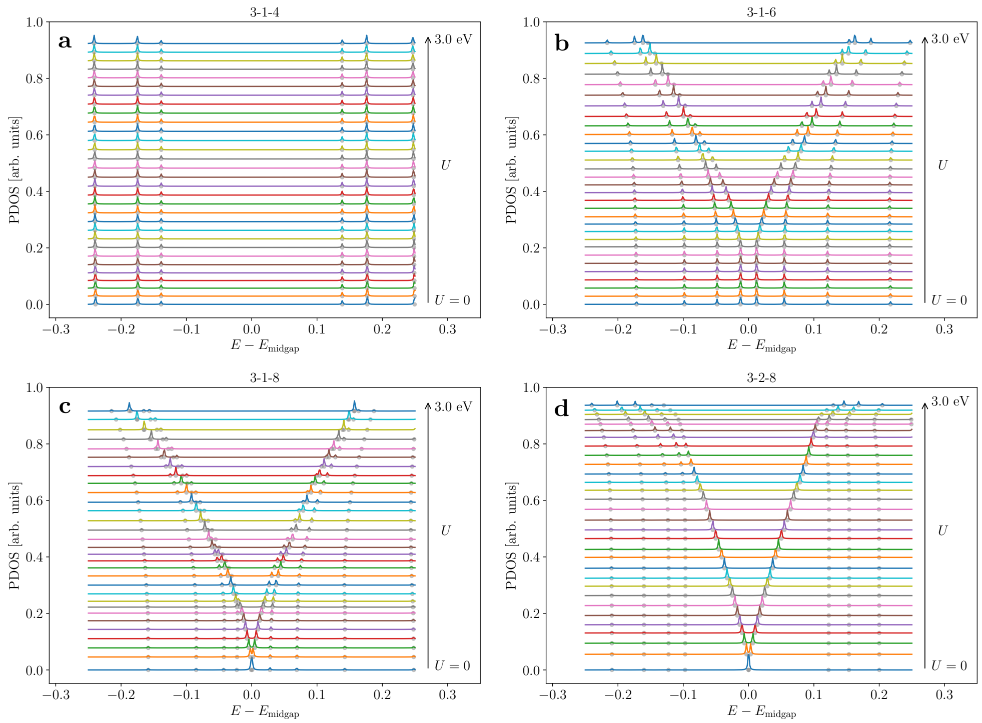

Supplementary Figure 23: Neutral systems: PDOS obtained at the atomic sites corresponding to where the end state localizes in the ribbon as a function of the electron energy and for different U values. For these calculations we use a Lorentzian smearing parameter $\eta = 1$ meV. The gray dots at the back of the plot indicate the eigenvalues of the system (independently if the state has weight at the end sites or not).

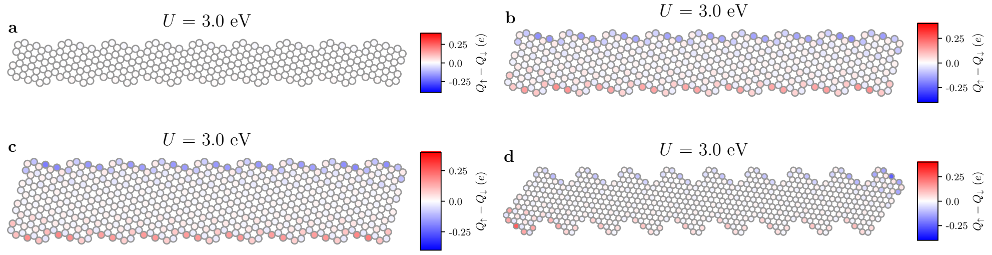

Supplementary Figure 24: Neutral systems: Spin polarizations for the finite molecule formed of 9 repetitions of the (n, m, w)-chGNR obtained with a strong Coulomb repulsion $U = 3$ eV.

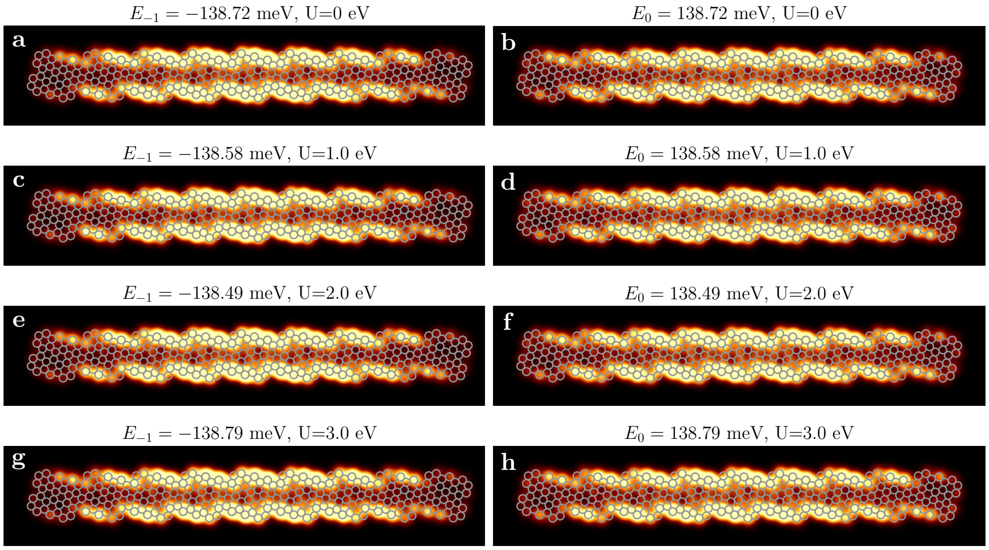

Supplementary Figure 25: Neutral systems: PDOS for the $(3, 1, 4)$ chGNR obtained at different U values. Lorentzian smearing of $\eta = 10$ meV is used, and the grid is sliced at $z = 5$ Å above the molecular plane. Left column is for occupied states while right column correspond to unoccupied states.

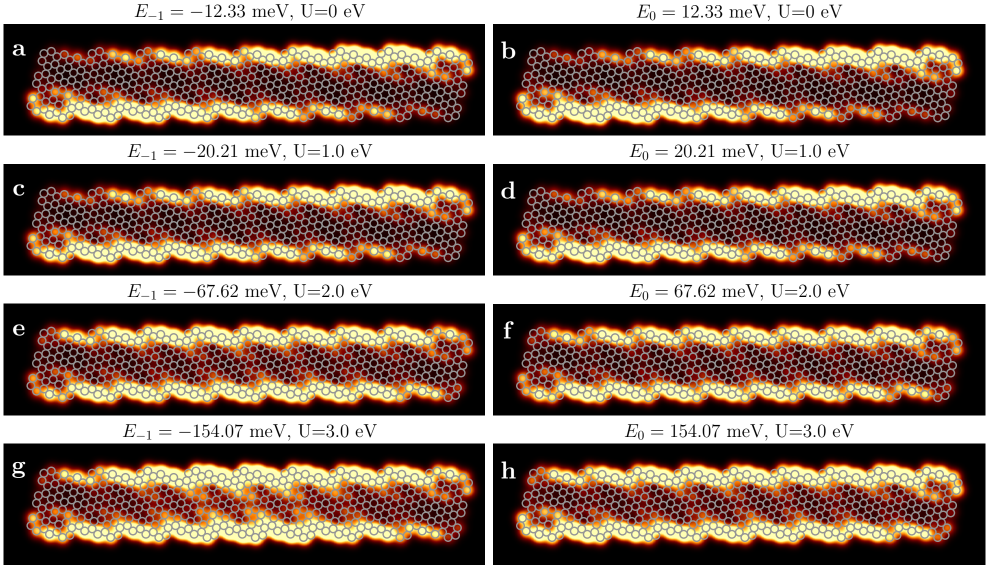

Supplementary Figure 26: Neutral system: PDOS for the $(3, 1, 6)$ chGNR, otherwise as Fig. 25.

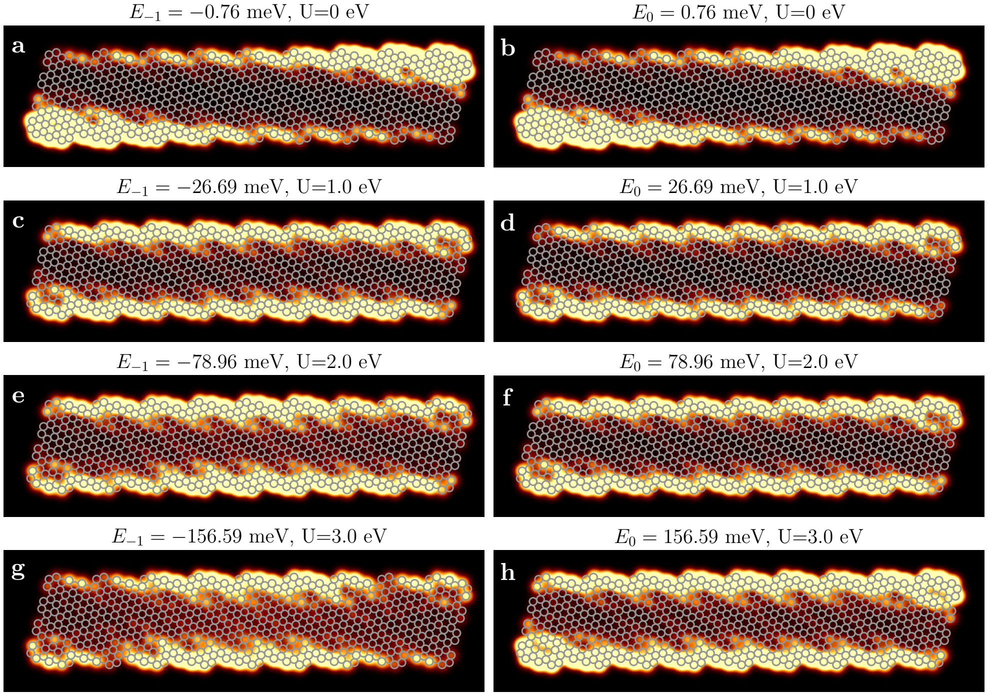

Supplementary Figure 27: Neutral system: PDOS for the $(3, 1, 8)$ chGNR, otherwise as Fig. 25.

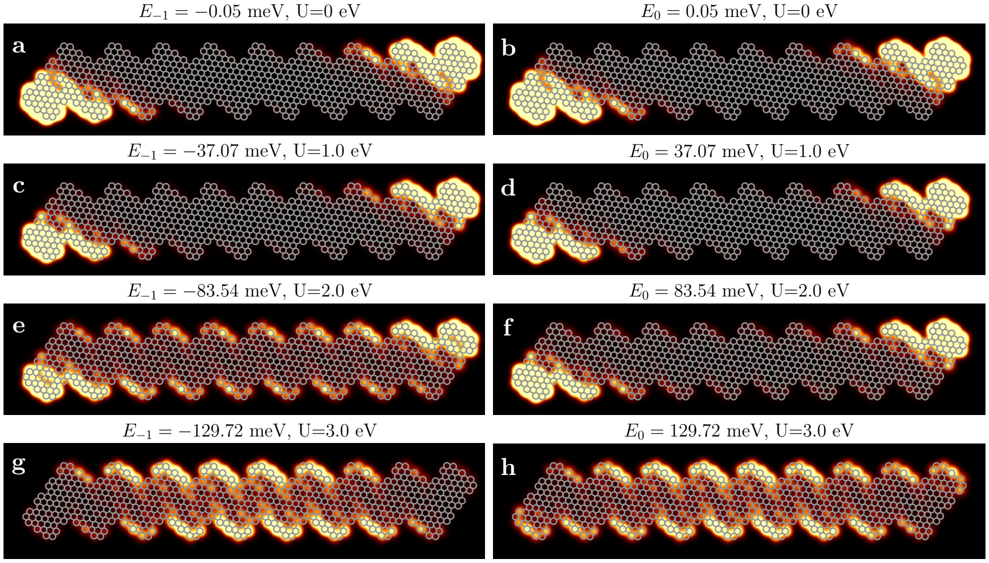

Supplementary Figure 28: Neutral system: PDOS for the $(3, 2, 8)$ chGNR, otherwise as Fig. 25.

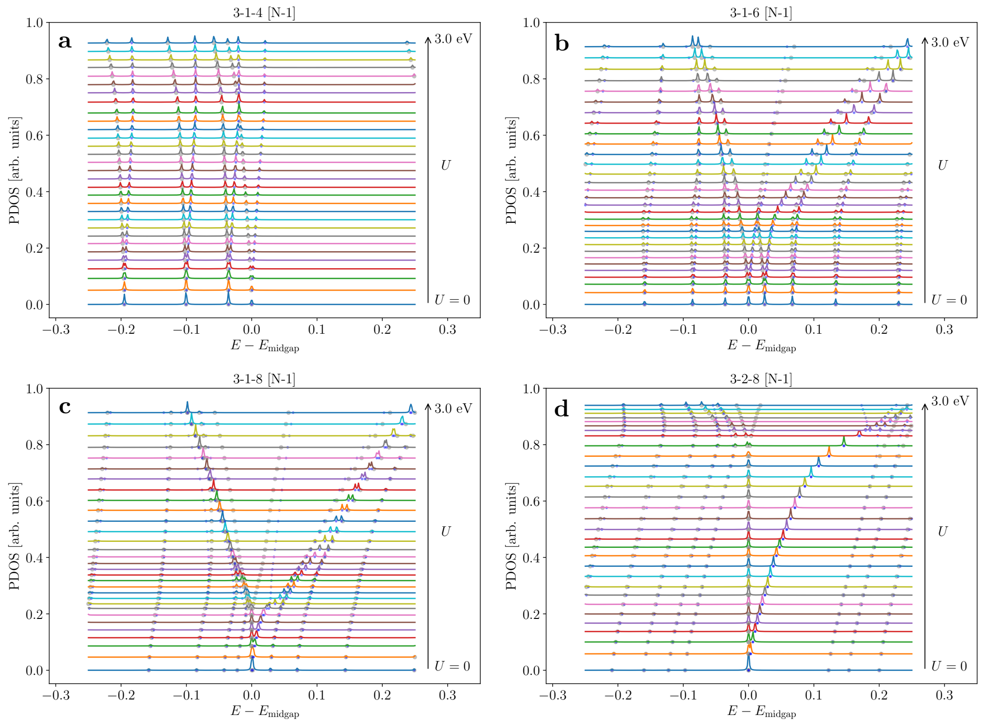

Supplementary Figure 29: Charged systems (one electron removed): PDOS obtained at the atomic sites corresponding to where the end state localizes in the ribbon as a function of the electron energy and for different U values. For these calculations we use a Lorentzian smearing parameter $\eta = 1$ meV. The gray (blue) dots at the back of the plot indicate the eigenvalues for up (down) electrons in the system (independently if the state has weight at the end sites or not).

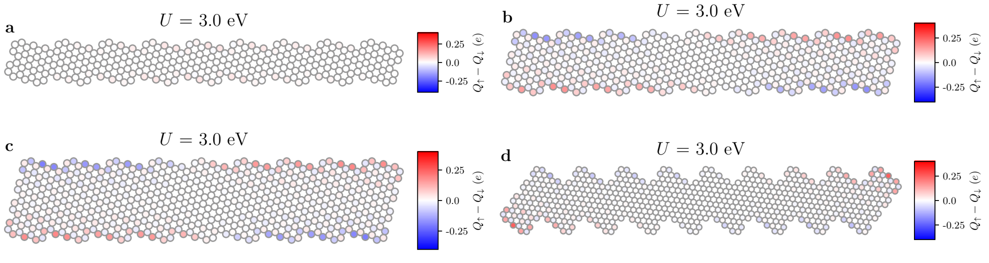

Supplementary Figure 30: Charged systems (one electron removed): Spin polarizations for the finite molecule formed of 9 repetitions of the (n, m, w)-chGNR obtained with a strong Coulomb repulsion $U = 3$ eV.

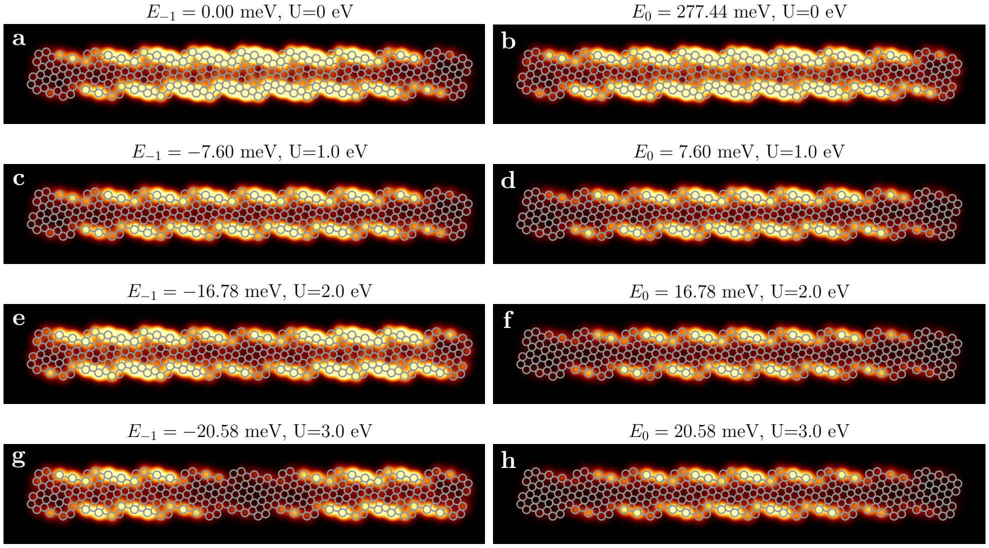

Supplementary Figure 31: Charged systems (one electron removed): PDOS for the $(3, 1, 4)$ chGNR obtained at different U values. A Lorentzian smearing of $\eta = 10$ meV is used, and the grid is sliced at $z = 5$ Å above the molecular plane. Left column is for occupied states while right column correspond to unoccupied states.

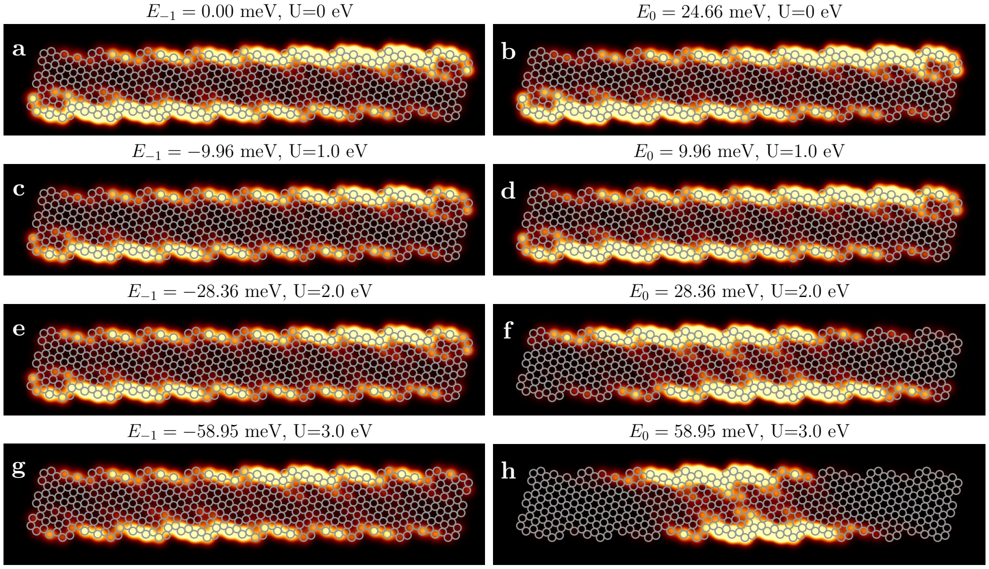

Supplementary Figure 32: Charged systems (one electron removed): PDOS for the $(3, 1, 6)$ chGNR, otherwise as Fig. 31.

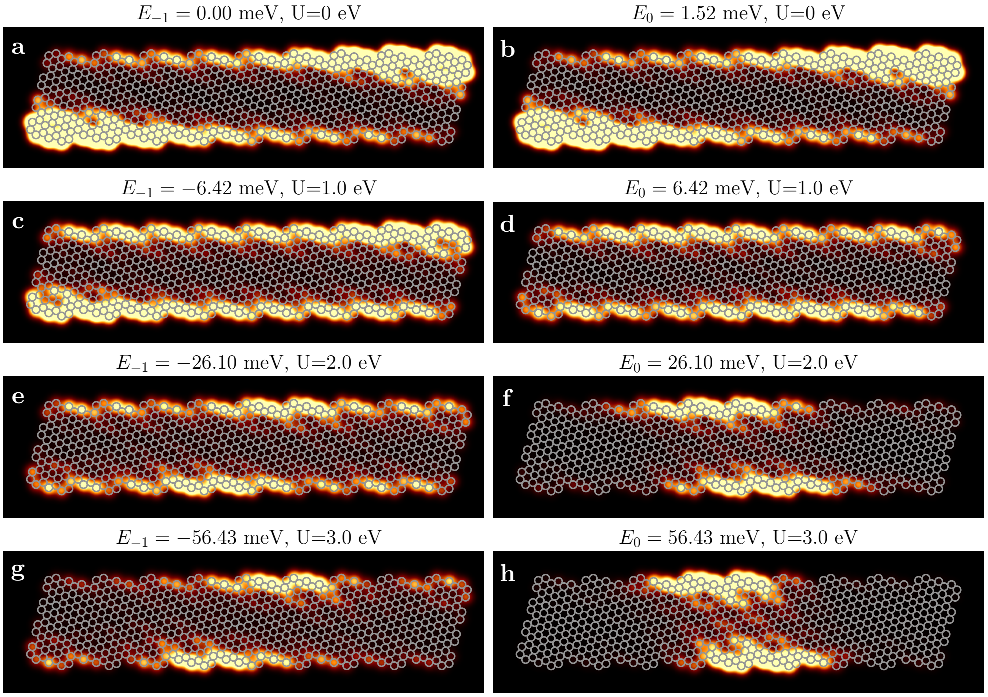

Supplementary Figure 33: Charged systems (one electron removed): PDOS for the $(3, 1, 8)$ chGNR, otherwise as Fig. 31.

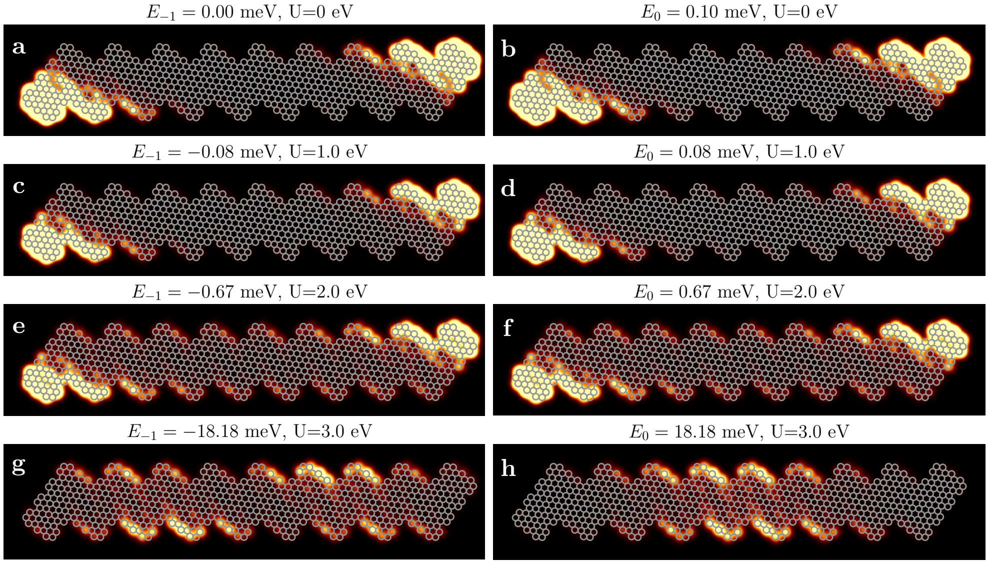

Supplementary Figure 34: Charged systems (one electron removed): PDOS for the $(3, 2, 8)$ chGNR, otherwise as Fig. 31.

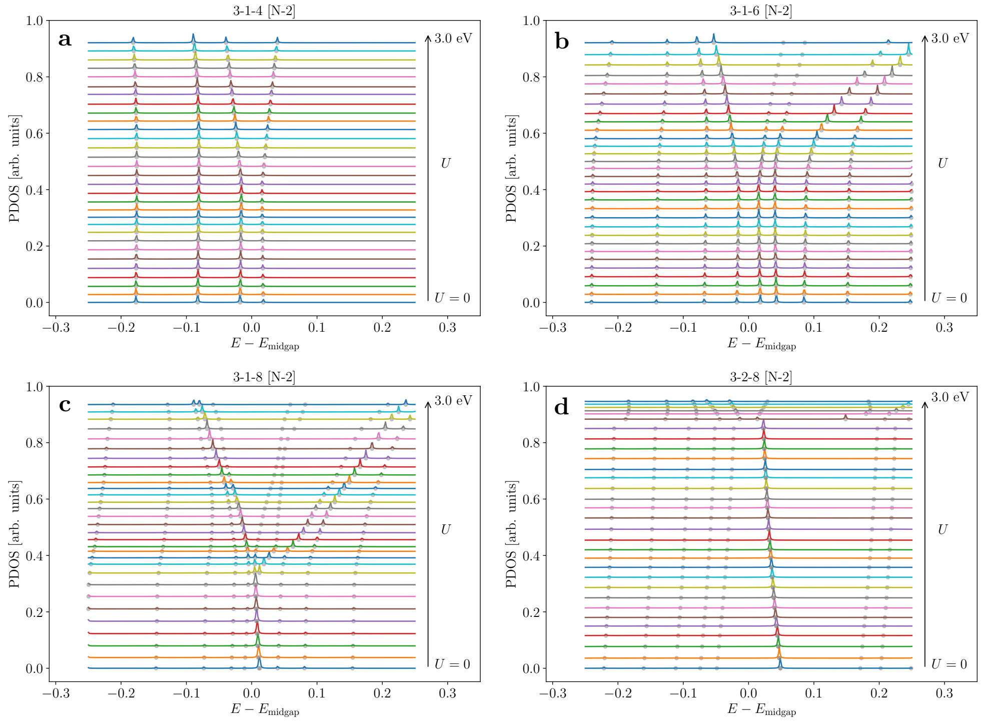

Supplementary Figure 35: Charged systems (two electrons removed): PDOS obtained at the atomic sites corresponding to where the end state localizes in the ribbon as a function of the electron energy and for different U values. For these calculations we use a Lorentzian smearing parameter $\eta = 1$ meV. The gray dots at the back of the plot indicate the eigenvalues of the system (independently if the state has weight at the end sites or not).

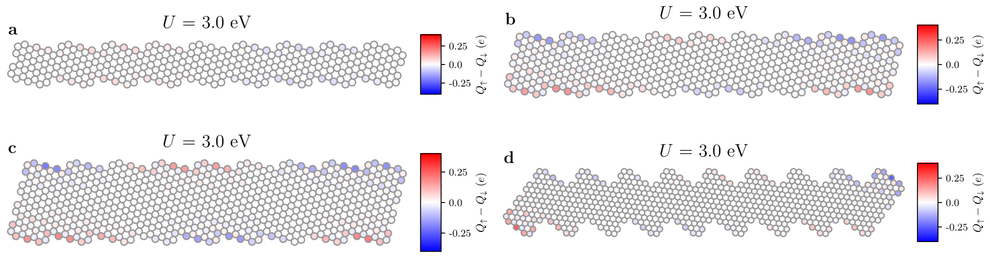

Supplementary Figure 36: Charged systems (two electrons removed): Spin polarizations for the finite molecule formed of 9 repetitions of the (n, m, w)-chGNR obtained with a strong Coulomb repulsion $U = 3$ eV.

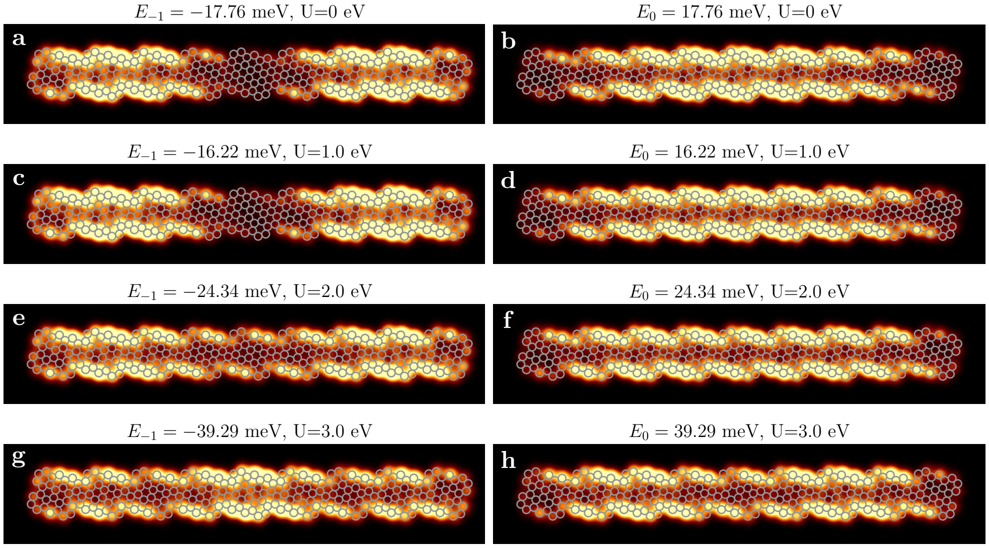

Supplementary Figure 37: Charged systems (two electrons removed): PDOS for the $(3, 1, 4)$ chGNR obtained at different U values. A Lorentzian smearing of $\eta = 10$ meV is used, and the grid is sliced at $z = 5$ Å above the molecular plane. Left column is for occupied states while right column correspond to unoccupied states.

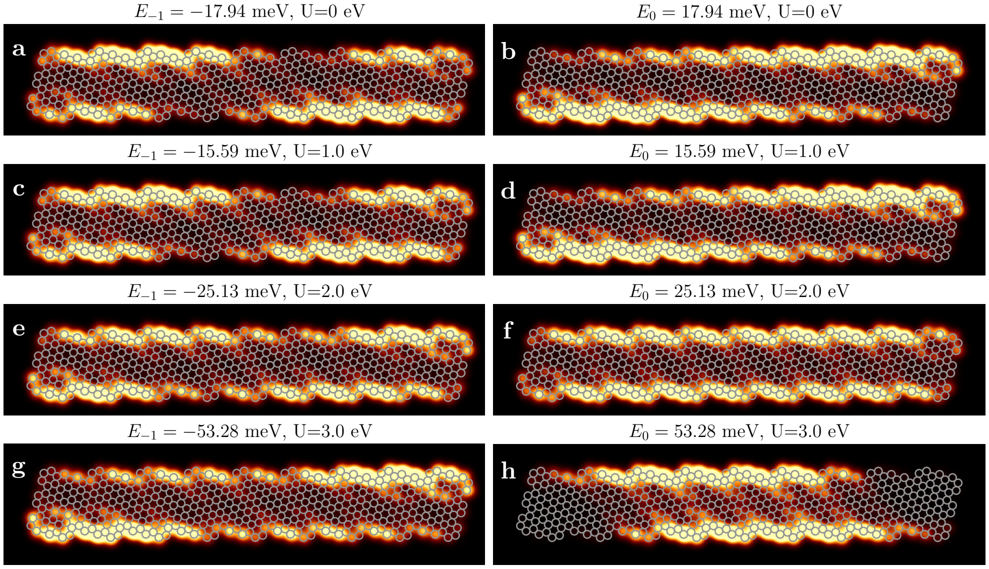

Supplementary Figure 38: Charged systems (two electrons removed): PDOS for the $(3, 1, 6)$ chGNR, otherwise as Supplementary Fig. 37.

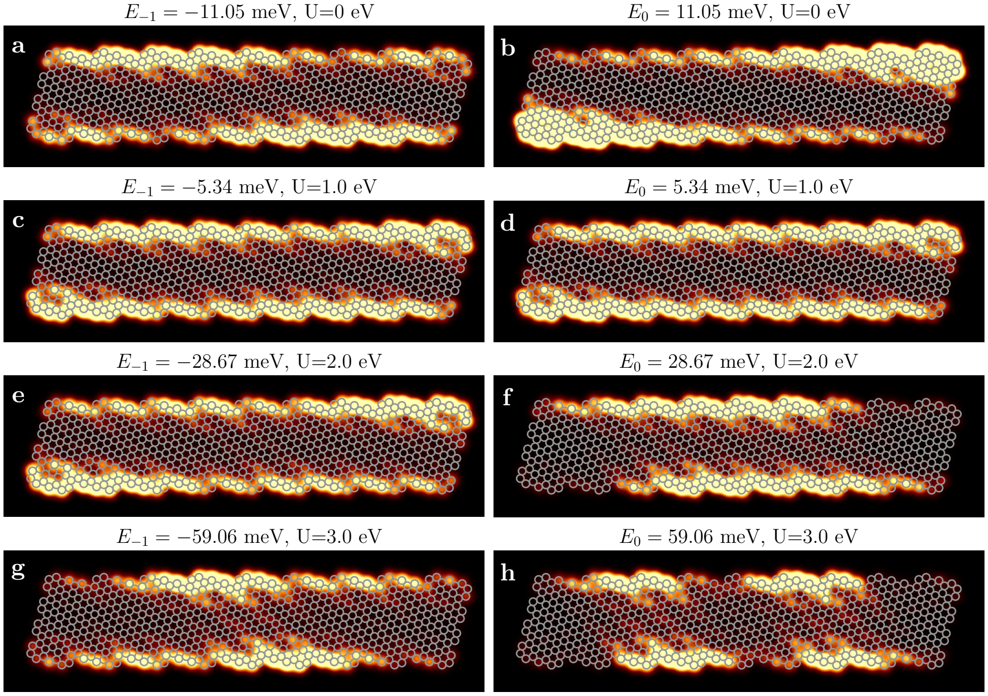

Supplementary Figure 39: Charged systems (two electrons removed): PDOS for the $(3, 1, 8)$ chGNR, otherwise as Supplementary Fig. 37.

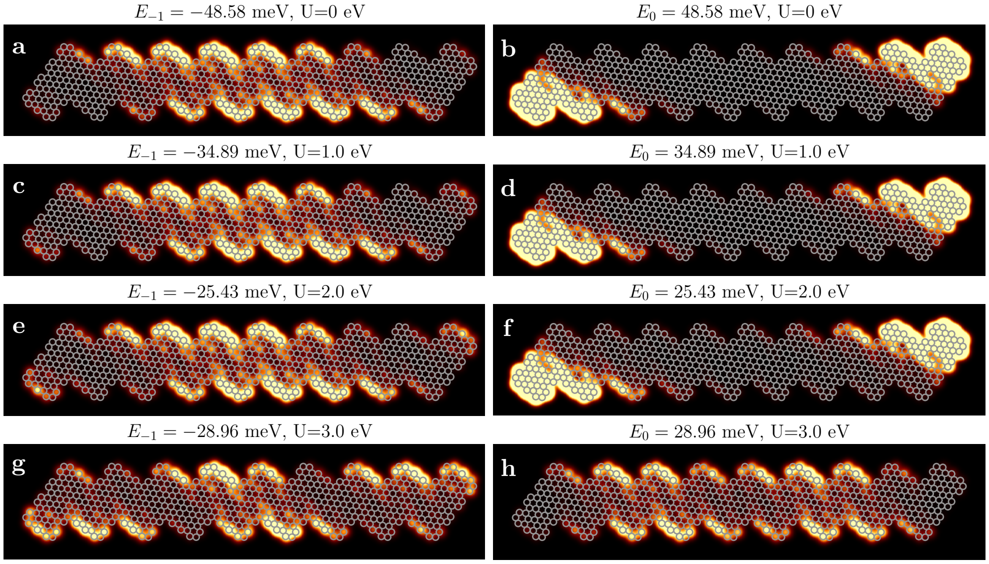

Supplementary Figure 40: Charged systems (two electrons removed): PDOS for the $(3, 2, 8)$ chGNR, otherwise as Supplementary Fig. 37.

References

1. Lin, Y.-Z. *et al.* Anthracene based organic dipolar compounds for sensitized solar cells. *Tetrahedron* **70**, 262 (2014).

2. Itoh, T., Matsuno, M., Kamiya, E., Hirai, K. & Tomioka, H. Preparation of Copper Ion Complexes of Sterically Congested Diaryldiazomethanes Having a Pyridine Ligand and Characterization of Their Photoproducts. *Journal of the American Chemical Society* **127**, 7078 (2005).

3. De Oteyza, D. G. *et al.* Substrate-Independent Growth of Atomically Precise Chiral Graphene Nanoribbons. *ACS Nano* **10**, 9000 (2016).

4. Hancock, Y., Uppstu, A., Saloriutta, K., Harju, A. & Puska, M. J. Generalized tight-binding transport model for graphene nanoribbon-based systems. *Phys. Rev. B* **81**, 245402 (2010).

5. Zak, J. Berry's phase for energy bands in solids. *Phys. Rev. Lett.* **62**, 2747 (1989).

6. Fu, L. & Kane, C. L. Topological insulators with inversion symmetry. *Phys. Rev. B* **76**, 045302 (2007).

7. Cao, T., Zhao, F. & Louie, S. G. Topological Phases in Graphene Nanoribbons: Junction States, Spin Centers, and Quantum Spin Chains. *Phys. Rev. Lett.* **119**, 76401 (2017).

8. Resta, R. Manifestations of berry's phase in molecules and condensed matter. *Journal of Physics: Condensed Matter* **12**, R107 (2000).

9. Papior, N. R. sisl: v0.11.0 (2021).

10. Merino-Díez, N. *et al.* Unraveling the Electronic Structure of Narrow Atomically Precise Chiral Graphene Nanoribbons. *The Journal of Physical Chemistry Letters* **9**, 25 (2018).

11. Gröning, O. *et al.* Engineering of robust topological quantum phases in graphene nanoribbons. *Nature* **560**, 209 (2018).

12. Kimouche, A. *et al.* Ultra-narrow metallic armchair graphene nanoribbons. *Nature Communications* **6**, 10177 (2015).

13. Merino-Díez, N. *et al.* Width-dependent band gap in armchair graphene nanoribbons reveals fermi level pinning on au(111). *ACS Nano* **11**, 11661 (2017).

14. Carbonell-Sanromà, E. *et al.* Quantum Dots Embedded in Graphene Nanoribbons by Chemical Substitution. *Nano Lett.* **17**, 50 (2017).

15. Fernández-Rossier, J. & Palacios, J. J. Magnetism in Graphene Nanoislands. *Phys. Rev. Lett.* **99**, 177204 (2007).

16. Yazyev, O. V. Emergence of magnetism in graphene materials and nanostructures. *Reports on Progress in Physics* **73**, 56501 (2010).

17. Yazyev, O. V., Capaz, R. B. & Louie, S. G. Theory of magnetic edge states in chiral graphene nanoribbons. *Phys. Rev. B* **84**, 115406 (2011).

18. Li, J. *et al.* Single spin localization and manipulation in graphene open-shell nanostructures. *Nat. Commun.* **10**, 200 (2019).

19. Li, J. *et al.* Uncovering the triplet ground state of triangular graphene nanoflakes engineered with atomic precision on a metal surface. *Phys. Rev. Lett.* **124**, 177201 (2020).
